# Parallel reductive genome evolution in *Desulfovibrio* ectosymbionts independently acquired by *Trichonympha* protists in the termite gut

**DOI:** 10.1038/s41396-020-0688-1

**Published:** 2020-06-01

**Authors:** Mariko Takeuchi, Hirokazu Kuwahara, Takumi Murakami, Kazuki Takahashi, Rei Kajitani, Atsushi Toyoda, Takehiko Itoh, Moriya Ohkuma, Yuichi Hongoh

**Affiliations:** 1 School of Life Science and Technology, Tokyo Institute of Technology, Tokyo, 152-8550 Japan; 2 Department of Informatics, National Institute of Genetics, Shizuoka, 411-8540 Japan; 3 Advanced Genomics Center, National Institute of Genetics, Shizuoka, 411-8540 Japan; 4 Department of Genomics and Evolutionary Biology, National Institute of Genetics, Shizuoka, 411-8540 Japan; 5 Japan Collection of Microorganisms, RIKEN BioResource Research Center, Tsukuba, 305-0074 Japan

**Keywords:** Next-generation sequencing, Molecular evolution, Comparative genomics, Bacterial evolution, Symbiosis

## Abstract

Several *Trichonympha* protist species in the termite gut have independently acquired *Desulfovibrio* ectosymbionts in apparently different stages of symbiosis. Here, we obtained the near-complete genome sequence of *Desulfovibrio* phylotype ZnDsv-02, which attaches to the surface of *Trichonympha collaris* cells, and compared it with a previously obtained genome sequence of ‘*Candidatus* Desulfovibrio trichonymphae’ phylotype Rs-N31, which is almost completely embedded in the cytoplasm of *Trichonympha agilis*. Single-nucleotide polymorphism analysis indicated that although Rs-N31 is almost clonal, the ZnDsv-02 population on a single host cell is heterogeneous. Despite these differences, the genome of ZnDsv-02 has been reduced to 1.6 Mb, which is comparable to that of Rs-N31 (1.4 Mb), but unlike other known ectosymbionts of protists with a genome similar in size to their free-living relatives. Except for the presence of a lactate utilization pathway, cell-adhesion components and anti-phage defense systems in ZnDsv-02, the overall gene-loss pattern between the two genomes is very similar, including the loss of genes responsive to environmental changes. Our study suggests that genome reduction can occur in ectosymbionts, even when they can be transmitted horizontally and obtain genes via lateral transfer, and that the symbiont genome size depends heavily on their role in the symbiotic system.

## Introduction

Termites efficiently digest lignocellulose in association with gut microbes [1, 2]. The microbial community comprises protists, bacteria, archaea, and viruses, which are unique to termites [3–7]. A prominent feature of this microbiota is a cellular association between protists and prokaryotes, which plays a significant role in the nutrition of phylogenetically basal (so-called ‘lower’) termites [1, 2, 8, 9].

One example of the above phenomenon is the association of several bacterial lineages with the cellulolytic protist genus *Trichonympha* (phylum *Parabasalia*). Most *Trichonympha* species obligatorily harbor ‘*Candidatus* Endomicrobium trichonymphae’ (phylum *Elusimicrobia*) [10–12] or ‘*Candidatus* Ancillula trichonymphae’ (phylum *Actinobacteria*) [13] in the cytoplasm. They are also facultatively associated with ectosymbiotic bacteria, including members of the order *Bacteroidales* [14] and genus *Treponema* [14, 15]. In addition to these obligate and facultative symbionts, several *Trichonympha* species are also associated obligatorily with endosymbiotic ‘*Candidatus* Adiutrix intracellularis’ [16] and/or ectosymbiotic ‘*Candidatus* Desulfovibrio trichonymphae’ [13, 16, 17], both of which belong to the class *Deltaproteobacteria*.

Although none of these symbiotic bacteria have been cultured, previous genomic analyses suggested functions for several of the species. For example, ‘*Ca*. Endomicrobium trichonymphae’ and ‘*Ca*. Ancillula trichonymphae’ play a role in upgrading nitrogenous compounds [18–20]. ‘*Ca*. Adiutrix intracellularis’ is capable of reductive acetogenesis using H_2_ and CO_2_ and can fix dinitrogen [16]. ‘*Ca*. Desulfovibrio trichonymphae’ phylotype Rs-N31 can oxidize hydrogen via sulfate and/or fumarate respiration and may contribute to the removal of hydrogen that can inhibit cellulose fermentation in the termite gut [21, 22].

Interestingly, different phylotypes of *Desulfovibrio* ectosymbionts exhibit different manners of association with their respective *Trichonympha* host species. Whereas phylotype ZnDsv-02 (or ZnvTc-7) (AB444082 and JQ993542) attaches to a shallow groove on the surface of *Trichonympha collaris* cells in the gut of the termite *Zootermopsis nevadensis* [16], phylotype ImrTc-15 (JQ993569) is held in a deep invagination on the surface of *Trichonympha globulosa* in the gut of *Incisitermes marginipennis* [13]. The localization of phylotype Rs-N31 provides a more unusual example for an ectosymbiont: its cells are almost completely embedded in the surface of *Trichonympha agilis* in the gut of *Reticulitermes speratus*, and some Rs-N31 cells can be observed deep within the host cell cytoplasm. Each Rs-N31 cell is connected to the outside of the host cell only by a pore or tube-like structure of ca. 40-nm diameter. The genome is as small as 1.4 Mb, which is only half or less of the genome size of its free-living relatives [21]. Thus, Rs-N31 appears to be an ‘almost intracellular’ symbiont [17, 21]. Rs-N31 cells are coccoid or oval shaped [17, 21], whereas ZnDsv-02 and ImrTc-15 cells are rod shaped [13, 16], more similar to free-living relatives. Phylogenetic analyses of 16S rRNA genes suggested that these three phylotypes were most likely independently acquired by the respective *Trichonympha* host lineages [13, 21]. Differences in the manner of association and cell morphology between these three phylotypes might be indicative of the depth of the interrelationship with the *Trichonympha* hosts and the length of time after acquisition as an ectosymbiont.

In this study, we obtained the near-complete sequence of the *Desulfovibrio* phylotype ZnDsv-02 genome and compared it with known sequences of the genomes of phylotype Rs-N31 and free-living relatives. Our analyses provide evidence of parallel reductive genome evolution of these ectosymbionts and insights into the evolutionary process from free-living to ectosymbiotic and further to an ‘almost intracellular’ lifestyle.

## Materials and methods

### Termites and protists

*Z. nevadensis* (family Archotermopsidae) were collected in Hyogo Prefecture, Japan, and kept in the laboratory by feeding pinewood chips [23] (Supplementary methods). The hindgut contents of a worker termite were suspended in sterile Trager’s solution U [24] containing 0.05% bovine serum albumin, and then cells of four *Trichonympha* species (*T. collaris*, *T. sphaerica*, *T. campanula*, and *T. postcylindrica*) were collected using an AM6000 inverted micromanipulation microscope system (Leica Microsystems, Wetzlar, Germany). *Trichonympha* species were identified based on both morphological characteristics and the 18S rRNA gene sequence [25, 26]. Other termite species used for fluorescence in situ hybridization (FISH) or transmission electron microscopy (TEM) were collected as described in the Supplementary methods.

### FISH and TEM

FISH analyses were performed as described previously [27, 28]. Probe DSV698 [29] was used in order to detect a broad range of *Desulfovibrio* bacteria. Probe ZnDsv-02-471, which is specific to *Desulfovibrio* phylotype ZnDsv-02 [17], was used in this study with newly designed helper probes (Table S1). Co-existing ‘*Ca*. Endomicrobium trichonymphae’ and ‘*Ca*. Adiutrix intracellularis’ were detected using specific probes designed in previous studies [16, 25] (Table S1). Cells were observed under a BX51 epifluorescence microscope or FV1000D-IX81 confocal laser scanning microscope (Olympus, Tokyo, Japan). TEM of *T. collaris* was performed as described previously [28, 30] by collecting 25 *T. collaris* cells using the micromanipulation system. TEM of *T. agilis* from *R. speratus* was performed as described previously [21].

### 16S rRNA amplicon sequencing analysis

To analyse the taxonomic composition of bacteria associated with single *Trichonympha* cells, the V3–V4 region (ca. 400 bp) of the 16S rRNA gene was amplified by PCR using a *Bacteria*-specific 341F and 785R primer set (Table S2) and DNA samples prepared by whole-genome amplification (WGA) (see below). The PCR products were subjected to sequencing on an Illumina MiSeq platform as described previously [31]. The sequence reads were trimmed, quality filtered and sorted into amplicon sequence variants (ASVs) using DADA2 v1.12.1 [32, 33] and phylogenetically classified using SINA v1.2.11 [34] with database SILVA SSURef NR99 release 132 [35], as described previously [36]. ASVs with <5 reads were discarded from the analysis.

To examine the abundance and phylogenetic diversity of the genus *Desulfovibrio* in the gut of various termite and cockroach species, the amplicon sequence datasets (BioProject PRJDB8349) of the 16S rRNA V3–V4 region analyzed in our previous study were used [36]. The sequence reads were sorted into ASVs in the same way described above, except that DADA2 v1.4 was used.

### Preparation of DNA from single protist cells and sequencing of the 18S rRNA gene

A single *T. collaris* cell was dissected into the anterior part that contained *Desulfovibrio* cells and the posterior part that contained the host nucleus, as described previously [21]. Both parts were collected separately and subjected to WGA using the illustra GenomiPhi V2 Kit (GE Healthcare, Chicago, IL, USA) as described previously [37]. The WGA product from the anterior part was subjected to a second WGA using the illustra GenomiPhi HY Kit (GE Healthcare) as described previously [38]. The resulting product was debranched using RepliPHI Phi29 DNA Polymerase (Epicentre, Madison, WI, USA) and then treated with S1 nuclease (Takara Bio, Shiga, Japan) [39]. To phylogenetically identify the host *Trichonympha* species, 18S rRNA genes amplified by PCR from WGA samples of the posterior part or whole cells were cloned and sequenced according to the Sanger method as described previously [40] (Supplementary methods).

### Genome sequencing, assembly, and binning

Sequencing libraries for the ZnDsv-02 genome were prepared using the TruSeq DNA PCR-Free Library Prep Kit and the Nextera Mate Pair Library Prep Kit (Illumina, Madison, WI, USA). Sequencing was performed using the MiSeq Reagent Kit v3 (600 cycles) on the Illumina MiSeq platform. After adapter removal and quality trimming using cutadapt [41] and prinseq [42], respectively, the resulting reads were assembled into contigs using SPAdes 3.10.1 [43]. The contigs and mate-pair reads were used to generate scaffolds with SCARPA 0.241 [44]. Contigs and scaffolds exhibiting the highest similarity to genome sequences of the genus *Desulfovibrio* or ‘*Ca*. Adiutrix intracellularis’ Adiu1 (LQAA00000000) by BLASTn searching against the NCBI non-redundant (nr) nucleotide database were extracted and treated as ‘trusted contigs.’ Sequence reads of 2–5 kb in length, which were obtained on a PacBio RSII platform as described previously [4], were added and re-assembled with the ‘trusted contigs’ using SPAdes 3.10.1. The resulting assemblies were manually inspected and curated as needed. After these processes, the *Desulfovibrio* genome was still fragmented to 55 contigs. Thus, we incorporated an assembling process as described in the Supplementary methods, and the final contig set was then obtained.

### Gene finding, annotation, and comparative genomics

Automatic gene finding and annotation were performed using MiGAP (http://www.migap.org/), RNAmmer 1.2 [45] and tRNAscan-SE 2.0 [46]. By conducting BLASTp searches against the NCBI nr protein database, the gene loci and annotations were manually curated. Pseudogenes were manually identified as described previously [18]. Among 139 single-copy marker genes proposed by Rinke et al. [47], 132 were conserved in *Desulfovibrio* and used to estimate genome completeness (Dataset S1). Metabolic pathways were predicted using the KEGG automatic annotation server [48]. Clustered regularly interspaced short palindromic repeat (CRISPR) loci were identified using CRISPRCasFinder [49]. Genes associated with secretion systems were detected using TXSScan [50]. Orthologous genes were detected using DomClust [51] with manual corrections. Genes and pseudogenes were classified into non-supervised orthologous groups (NOGs) using EggNOG 4.5.1 [52]. The absence of genes that are functionally important but missing in the ZnDsv-02 genome were re-confirmed by conducting tBLASTn searches against the contigs not included in the ZnDsv-02 assembly, using the amino acid sequences of relatives as queries.

### Detection and comparison of single-nucleotide polymorphisms (SNPs) and indels

Quality-filtered MiSeq paired-end reads were mapped to genome sequences using Burrows-Wheeler Aligner 0.7.17 [53]. After removing duplicated reads using SAMtools [54], the average read depth was calculated using DepthOfCoverage implemented in the Genome Analysis Tool Kit (GATK) 3.8.1.0 [55]. The outputs were downsized using DownsampleSam in GATK 4.1.2.0 to adjust the average read depth among samples. Sequence variants were identified using HaplotypeCaller implemented in GATK 4.1.2.0. Reads with mapping quality <30 were eliminated from the analysis. The resulting variants with a read depth of <10 were discarded and then annotated using snpEff 4.3 [56].

### Phylogenetic analysis, average nucleotide identity (ANI), and average amino acid identity (AAI)

Nucleotide or amino acid sequences were aligned using MUSCLE [57] with manual corrections, and ambiguously aligned sites were removed using Gblocks [58]. Maximum-likelihood (ML) trees were inferred using MEGA6 [59] or MEGA7 [60]. Amino acid or nucleotide substitution models were selected using model test implemented in MEGA. The robustness of tree topologies was evaluated by 500 bootstrap resamplings. Bayesian trees were also constructed using MrBayes 3.2.1 [61] with four Markov chains running simultaneously for 1,000,000 generations. An ML tree of the 16S rRNA V3–V4 region was inferred, using IQ-TREE 1.6.11 [62] with the ModelFinder option. In this analysis, an ML tree based on near full-length 16S rRNA gene sequences was used as the constraint tree, and the confidence values were calculated using the ultrafast bootstrap approximation and SH-like approximate likelihood ratio test (1000 replicates). ANI and AAI were calculated using the ANI/AAI calculator [63, 64].

## Results

### Distribution of *Desulfovibrio* phylotype ZnDsv-02 in the gut of *Z. nevadensis*

**Fig. 1:**
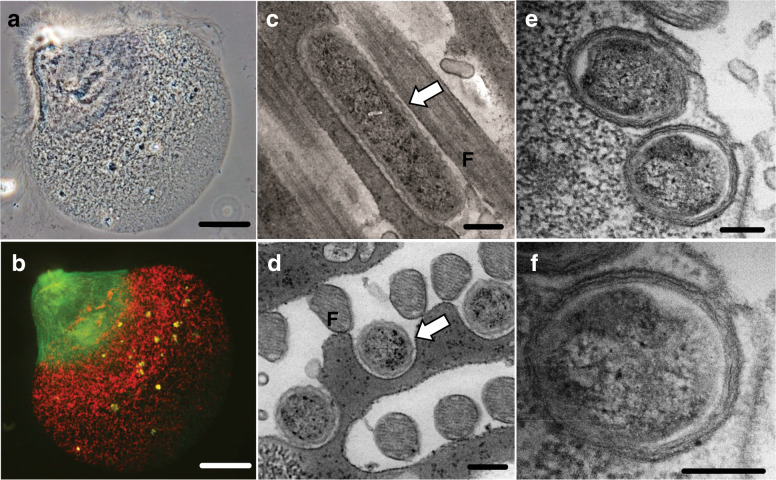
Localization of *Desulfovibrio* phylotype ZnDsv-02 and comparison with phylotype Rs-N31. **a** Phase-contrast image of *Trichonympha collaris* from the gut of *Zootermopsis nevadensis*. **b** Fluorescence in situ hybridization using oligonucleotide probes specific to ZnDsv-02 (6FAM-labeled, green) and ‘*Ca*. Endomicrobium trichonymphae’ (Texas red-labeled, red) (Table S1). The epifluorescent images were merged. **c**, **d** Transmission electron microscopy (TEM) of ZnDsv-02 cells. **e**, **f** TEM of Rs-N31 cells. **F** Host’s flagella. Bars: (**a**, **b**), 50 μm; (**c**–**f**), 200 nm.

Among the four *Trichonympha* species present in the gut of *Z. nevadensis*, *Desulfovibrio* were detected almost only in *T. collaris* cell samples by 16S rRNA amplicon sequencing analysis (Fig. S1a) and only in *T. collaris* cells by FISH analysis using probe DSV698 (data not shown). Almost all of the *Desulfovibrio* ASVs obtained from the *T. collaris* cells were identical to (ASV_T4) or differing by one base (ASV_T8) from the ZnDsv-02 sequence (AB444082) (Fig. S1b). ZnDsv-02 cells were specifically detected by FISH using probe ZnDsv-02-471 in the anterior part of *T. collaris* cells with co-existing ‘*Ca*. Endomicrobium trichonymphae’ in the posterior part (Fig. 1a, b). ‘*Ca*. Adiutrix intracellularis’ cells were also detected by FISH in both the anterior and posterior parts of the host cells (Fig. S2). TEM demonstrated that ZnDsv-02 cells were held in hollow-like structures on the host cell surface (Fig. 1c, d). Almost half of the ZnDsv-02 cell surface was exposed to the exterior, unlike the cells of Rs-N31 (Fig. 1e, f) [21]. These results were consistent with the previous study using *Z. nevadensis* collected in North America [16].

FISH analyses using probe DSV698 also indicated the association of *Desulfovibrio* bacteria with *Trichonympha* species in the gut of *Reticulitermes yaeyamanus*, but not with *T. agilis* in the gut of *Reticulitermes flavipes*. No cellular association of *Desulfovibrio* was observed in the gut protistan community of *Coptotermes formosanus* and *Neotermes koshunensis* (data not shown).

### Reconstruction and general features of the genome of *Desulfovibrio* phylotype ZnDsv-02

We reconstructed a draft genome sequence of *Desulfovibrio* phylotype ZnDsv-02 from a single *T. collaris* cell in a *Z. nevadensis* gut (Table 1) and simultaneously determined the complete genome sequence of ‘*Ca*. Adiutrix intracellularis’ (data not shown). We designated this ‘*Ca*. Adiutrix intracellularis’ genome sequence as genomovar Adiu2019 to discriminate it from the draft genome obtained in the previous study [16]. In addition, the complete genome sequence of a bacterium belonging to a clade of the order *Mycoplasmatales*, specific to the termite gut microbiota [65], was reconstructed. Details of these two genomes will be published elsewhere. The ratio of sequence reads mapped to each of the three genome sequences was described in the Supplementary results.

**Table 1:** Comparison of general genome features of *Desulfovibrio* phylotype ZnDsv-02, ‘*Ca* Desulfovibrio trichonymphae’ phylotype Rs-N31 and their free-living relatives.

	*Desulfovibrio* phylotype ZnDsv-02	‘*Ca*. Desulfovibrio trichonymphae’ Rs-N31	*D. fairfieldensis* CCUG45958	*D. desulfuricans* ATCC27774
Lifestyle	Surface-attached symbiont	Surface-embedded symbiont	Free-living	Free-living
Status (completeness)^a^	Draft (43 contigs) (99.2%)	Complete	Complete	Complete
Genome size (Mb)	1.6 (estimated)	1.4	3.7	2.9
CDS	1 257	1 082	3 022	2 356
G + C (%)	53.6	54.8	60.9	58.1
rRNA genes	3	6	9	9
tRNA genes	49	49	54	52
Pseudogenes	86	188	–	–
Coding density (%)	82.6	79.8	84.1	83.0

^a^Calculated based on single-copy marker genes (Dataset S1; see “Materials and methods”).

The ZnDsv-02 draft genome consisted of 43 contigs >300 bp (1 611 038 bp in total), and the estimated genome completeness was 99.2%. No contaminating contigs were identified based on CheckM [66] and manual inspection. The estimated genome size (1.6 Mbp) was much smaller than that of free-living relatives, such as *Desulfovibrio fairfieldensis* (3.7  Mbp) and *Desulfovibrio desulfuricans* (2.9 Mbp), but larger than that of ‘*Ca*. Desulfovibrio trichonymphae’ phylotype Rs-N31 (1.4 Mbp). The largest contig was 232 244 bp, and the N50 value was 90 504 bp. The G + C content (53.6%) was similar to that of Rs-N31 (54.8%), and these were among the lowest G + C contents of known *Desulfovibrio* species, apart from *Desulfovibrio hydrothermalis* AM13 (45.1%) and *Desulfovibrio salexigens* DSM 2638 (47.1%) (Fig. S3). Of 1257 putative protein-coding genes predicted (Dataset S2), 176 returned the highest BLASTp scores to genes of non-*Desulfovibrio*/*Lawsonia*/*Bilophila* bacteria (Table S3). The draft genome contained a single set of the rRNA gene operon, 49 tRNA genes for all amino acids, and 86 pseudogenes. One CRISPR-Cas system was found, with 309 spacer sequences (Dataset S3).

### Identification of SNPs and indels

As we were not able to reconstruct the complete genome sequence of ZnDsv-02, we hypothesized that the ZnDsv-02 population on a single host cell was heterogeneous. We therefore estimated the number of SNPs and indels in the ZnDsv-02 and Adiu2019 population genomes as well as those of Rs-N31 [21] and ‘*Ca*. Endomicrobium trichonymphae’ phylotype Rs-D17 genomovar Ti2015 [19]. When the average read depth was adjusted to the lowest value, 204, the frequencies of the sum of SNPs and indels per kilobase were as follows: 16 (ZnDsv-02), 0.027 (Rs-N31), 0.80 (Adiu2019), and 0.77 (Rs-D17) (Table S4). To evaluate the possibility that only a few distinct genomovars in small amounts contributed to the SNPs in the ZnDsv-02 genome, we inspected each sequence read in two genome regions (length: 100 bp each, read depth: 50 and 41) (Table S5). In both regions, the mapped reads were sorted to eight and six sequence types with different SNP patterns, respectively, and several sequence types were found in similar frequencies (Table S5). Thus, it is likely that the ZnDsv-02 population on a single host cell consisted of several genomovars. The detection of two 16S rRNA ASVs from single *T. collaris* cells, differing by one base (Fig. S1b), may also be indicative of heterogeneity. Details of the SNP and indel analysis were described in the Supplementary results, Dataset S4 and Fig. S4.

### Phylogenomics, ANI, and AAI

A phylogenomic tree was constructed; phylotype ZnDsv-02 clustered with Rs-N31, *D. desulfuricans*, *Desulfovibrio* sp. G11, *D. fairfieldensis* and *D. piger* (Fig. S5). ZnDsv-02 shared <96% 16S rRNA sequence identity, <79% ANI and <71% AAI with each of these bacteria, including Rs-N31 (Table S6). Among them, *D. fairfieldensis* CCUG45958 and *D. desulfuricans* ATCC27774 were selected as representative free-living relatives for further comparisons.

### Comparative analysis of the abundance of genes classified by functional category

**Fig. 2:**
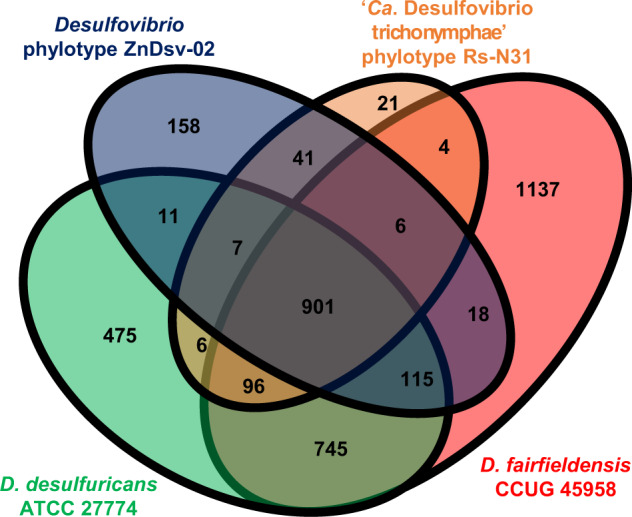
Core and pan-genome of *Desulfovibrio* phylotypes. The genomes of ZnDsv-02, Rs-N31 and their free-living relatives *Desulfovibrio fairfieldensis* and *Desulfovibrio desulfuricans* were compared.

**Fig. 3:**
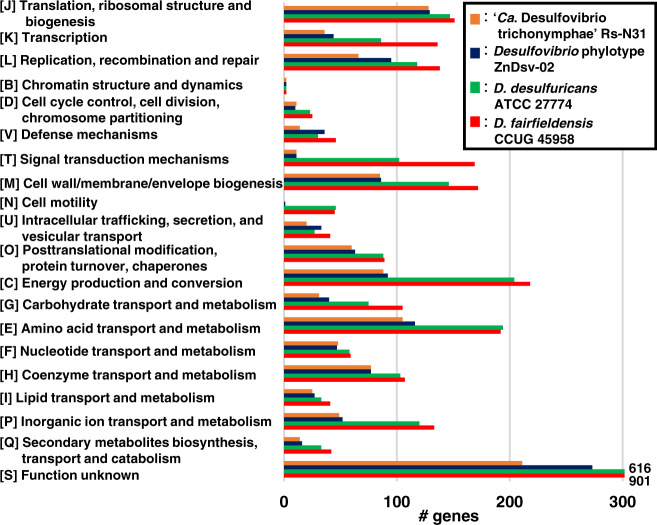
Comparison of the number of genes classified into non-supervised orthologous groups. The genomes of *Desulfovibrio* phylotypes ZnDsv-02 and Rs-N31, and their free-living relatives *Desulfovibrio fairfieldensis* and *Desulfovibrio desulfuricans* were compared.

The genomes of ZnDsv-02, Rs-N31, *D. fairfieldensis* and *D. desulfuricans* shared 901 orthologous genes, and 158, 21, 1137, and 475 genes were unique, respectively (Fig. 2, Dataset S5). The number of ZnDsv-02 genes in most NOGs was much fewer than in the free-living relatives and very similar to that of Rs-N31 (Fig. 3). This reduction in the number of genes was conspicuous, particularly in two categories: signal transduction mechanisms [T] and cell motility [N] (Figs. 3 and S6). In both the ZnDsv-02 and Rs-N31 genomes, many genes for response regulator proteins, chemotaxis and flagellar components are missing or pseudogenized; both ectosymbionts are likely nonmotile and have almost lost the ability to respond to environmental changes. Considerable differences were found between the ZnDsv-02 and Rs-N31 genomes in only a few categories: category [L], including DNA methyltransferase and CRISPR-associated proteins; [V], including DNA restriction enzymes; and [U], including the type IV secretion system (Figs. 3 and S6). The ZnDsv-02 genome contains 11 intact restriction-modification (RM) systems, in addition to 1 CRISPR-Cas system, whereas the Rs-N31 genome contains only pseudogenized RM and CRISPR-Cas systems. Among genes involved in DNA repair and recombination, both phylotypes have lost the genes encoding DnaQ, the proofreading 3′–5′ exonuclease subunit of the DNA polymerase III core, and formamidopyrimidine-DNA glycosylase (Fpg), which removes oxidized guanines and prevents the G-C to T-A transversion mutation (Table S7).

Pseudogenes found in the ZnDsv-02 genome were also classified into NOGs (Fig. S4 and Table S8). In most categories, the pseudogenes were more abundant in Rs-N31 than in ZnDsv-02. The number of pseudogenes in ZnDsv-02 exceeded that in Rs-N31 only in categories [J] and [H] (Fig. S4), including cobalamin biosynthesis proteins. The overall distribution pattern of pseudogenes among NOGs was significantly correlated between ZnDsv-02 and Rs-N31 (two-sided Spearman’s rank correlation test adjusted with Shaffer’s modified Bonferroni procedure, *r*_*s*_ = 0.55; *p* = 0.01).

To identify NOGs contributing to the difference in genome size between the *Desulfovibrio* ectosymbionts and ectosymbionts belonging to *Bacteroidales*, the total length of genes assigned to each NOG was compared (Fig. S7). Details were described in the Supplementary Results.

### Comparison of predicted metabolic pathways

**Fig. 4:**
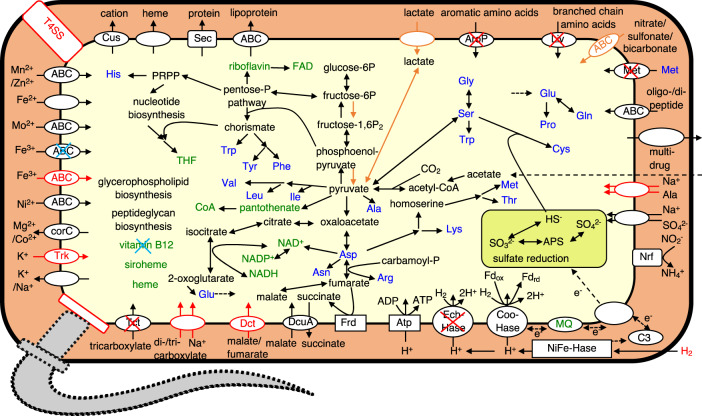
Comparison of predicted metabolic pathways between *Desulfovibrio* phylotypes ZnDsv-02 and Rs-N31. Pathways found in both phylotypes are shown in black; those found only in Rs-N31 are shown in black with a red ‘X’ mark; those found in Rs-N31, but pseudogenized in ZnDsv-02 are shown in black with a blue cross; those found only in ZnDsv-02 are shown in red; those found in ZnDsv-02, but pseudogenized in Rs-N31 are shown in orange. Flagellar components are absent in both phylotypes, except for fliM in ZnDsv-02.

The metabolic pathways of ZnDsv-02 were predicted and compared with those of Rs-N31 (Fig. 4). Both ZnDsv-02 and Rs-N31 retain pathways to synthesize 20 amino acids and various cofactors (Tables S9 and S10). Although we previously predicted that Rs-N31 cannot synthesize threonine and methionine [21], the gene RSDT_0983, annotated as one of two shikimate kinase genes, likely encodes a ‘missing’ homoserine kinase [67], and we later noted that the biosynthetic pathway from aspartate to methionine is complete in the Rs-N31 genome. Thus, the biosynthetic pathways for methionine and threonine are likely present in Rs-N31 as well as ZnDsv-02. Several genes in the ZnDsv-02 genome involved in cobalamin biosynthesis are either missing or pseudogenized. Although Rs-N31 possesses only a cobalamin-dependent methionine synthase gene (*metH*), ZnDsv-02 harbors genes for both MetH and cobalamin-independent methionine synthase MetE (Dataset S2).

The genomes of both ZnDsv-02 and Rs-N31 possess pathways for gluconeogenesis and non-oxidative pentose phosphate biosynthesis. The ZnDsv-02 genome retains the glycolytic pathway with the exception of lacking a glucokinase; thus, ZnDsv-02 likely does not utilize sugars, similar to Rs-N31, which has also lost 6-phosphofructokinase and pyruvate kinase [21]. The presence in the ZnDsv-02 genome of a lactate utilization pathway, which is pseudogenized in Rs-N31, suggests that lactate is used as a carbon and energy source. The tricarboxylic acid cycle is incomplete in both phylotypes; each lacks genes for malate dehydrogenase, succinate dehydrogenase and succinyl-CoA synthetase. These ectosymbionts probably cannot use succinate as a carbon source but may use malate and fumarate to produce L-aspartate via an aspartate-ammonia lyase. ZnDsv-02 retains the tripartite ATP-independent periplasmic high affinity transporter DctPQM, which probably imports malate and/or fumarate [68]. DctPQM is missing in Rs-N31, but a putative malate/succinate antiporter, DcuA, is encoded by both genomes. Both ectosymbionts retain the ability to use acetate and CO_2_ as carbon sources. Genes for the tricarboxylate transporter TctABC found in Rs-N31 are not present in the ZnDsv-02 genome, although the latter instead possesses a Na^+^-dependent di- or tricarboxylate symporter. Thus, both may use citrate or other carboxylates as carbon sources.

Genes involved in H_2_ oxidation in Rs-N31, including those encoding dissimilatory sulfite reductase, fumarate reductase (FrdABCD), F_o_F_1_-ATPase and several hydrogenases, were also found in the ZnDsv-02 genome, with the exception of the membrane-bound H^+^-translocating [NiFe] hydrogenase complex EchABCDEF (Table S11). For both ectosymbionts, DcuA and FrdABCD, required for fumarate respiration, exhibited the highest BLASTp scores with those of gammaproteobacteria in the NCBI nr protein database (Fig. S8). Neither phylotype retains the gene for the redox-sensing transcription factor Rex, which functions as a repressor of genes involved in sulfate reduction [69, 70]. Genes encoding catalase, superoxide dismutase and cytochrome *bd* are absent in the ZnDsv-02 genome, as in the Rs-N31 genome (Table S12). Overall, it is likely that ZnDsv-02 utilizes H_2_ and lactate as electron donors and sulfate and fumarate as electron acceptors to generate energy via anaerobic respiration.

Genes for nitrogen fixation and transporters for ammonium or urea are absent, suggesting that both ectosymbionts utilize amino acids and oligopeptides as primary nitrogen sources. The gene encoding the aromatic amino acid transporter AroP is absent in the ZnDsv-02 genome, which was found in the genomes of both Rs-N31 and ‘*Ca*. Endomicrobium trichonymphae’ Rs-D17 [21]. Phylotypes ZnDsv-02 and Rs-N31 possess the biosynthetic pathway for peptidoglycan, but they have lost several genes involved in lipopolysaccharide biosynthesis. Among phylotypes ZnDsv-02 and Rs-N31 and their two free-living relatives, only ZnDsv-02 possesses several components for a type IV secretion system, including VirB5, which may function as an adhesin, although the gene encoding VirB4, which functions as an ATPase and is likely essential for secretion and pilus assembly, is pseudogenized [71] (Table S13, Fig. S9).

### Phylogenetic diversity and abundance of bacteria belonging to *Desulfovibrio* ‘termite cluster I’

**Fig. 5:**
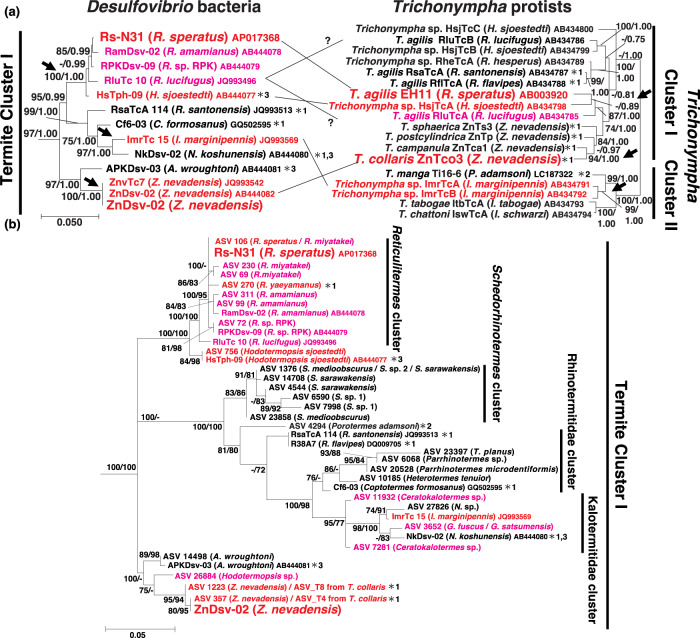
Phylogenetic trees of *Desulfovibrio* and *Trichonympha* based on small subunit (SSU) rRNA genes. The host termites are given in parentheses. Red: the association of *Desulfovibrio* and *Trichonympha* was confirmed by FISH; magenta: host termites harbor *Trichonympha* but the association was not examined by FISH; black: no association of *Desulfovibrio* and *Trichonympha* was observed. **a** A maximum-likelihood (ML) tree of *Desulfovibrio* (left) and *Trichonympha* (right) based on near full-length SSU rRNA genes, constructed using MEGA6 and MEGA7 with the K2 + G + I and GTR + G + I nucleotide substitution models, respectively. A total of 1165 and 1471 aligned nucleotides sites were used. Bootstrap confidence values (left; >70%) and posterior probabilities in Bayesian statistics (right; >0.90) are shown for the internal branches. Sequences used as the outgroup and omitted from the tree are listed in Table S14. Black arrows denote the predicted timing when the association of *Desulfovibrio* with *Trichonympha* has evolved. **b** An ML tree of *Desulfovibrio* based on 16S rRNA amplicon sequence variants, constructed using the TIM3 + F + I + G4 model in IQ-Tree. A total of 418 aligned nucleotide sites were used. SH-like approximate likelihood ratio test values (left; >70%) and ultrafast bootstrap confidence values (right; >70%) are shown. A: *Archotermopsis*, C: *Coptotermes*, G: *Glyptotermes*, H: *Hodotermopsis*, I: *Incisitermes*, N: *Neotermes*, P: *Porotermes*, R: *Reticulitermes*, S: *Schedorhinotermes*, T: *Termitogeton*, Z: *Zootermopsis*. *The cellular association of *Desulfovibrio* with *Trichonympha* was tested by FISH analysis: *1 in this study; *2 [15]; and *3 [17].

As indicated in previous studies [13, 17], the 16S rRNA genes of ZnDsv-02 and Rs-N31 clustered with those obtained exclusively from the termite gut, and the association with *Trichonympha* species has evolved independently at least three times (Fig. 5a, Table S14 and Supplementary results). To further explore the phylogenetic diversity of this monophyletic cluster (‘termite cluster I’ [17]), we searched our 16S rRNA amplicon datasets obtained from the gut microbiota of 62 termite and 10 cockroach species [36] for sequences assigned to this clade. ASVs from the gut of 22 ‘lower’ termite (non-Termitidae termites, with symbiotic gut protists) species were affiliated with ‘termite cluster I’ (Fig. 5b). No cockroach or ‘higher’ termite (family Termitidae, with no symbiotic gut protists) sequences were assigned to this clade. The 22 ‘lower’ termite species included termite genera that associate and do not associate with *Trichonympha*. In *Z. nevadensis, Hodotermopsis sjoestedti* and *Reticulitermes* species, in which the cellular association of *Desulfovibrio* symbionts with *Trichonympha* has been reported [13, 16, 17], the total frequency of ASVs in ‘termite cluster I’ ranged from 2.4 to 16.9% and constituted most of the *Desulfovibrio* sequences detected in the gut of the respective termite species (Table S15).

## Discussion

Our comparative genomic analysis of *Desulfovibrio* phylotype ZnDsv-02, ‘*Ca*. Desulfovibrio trichonymphae’ phylotype Rs-N31 and their free-living relatives sheds light on genomic evolution during the course of cellular symbiosis. Although ZnDsv-02 and Rs-N31 were independently acquired by the host *Trichonympha* lineages (Fig. 5a and Supplementary results), both genomes exhibited very similar reductive genome evolution, as exemplified by the number of genes in each functional category (Fig. 3). It is of particular interest that the number of genes related to signal transduction in ZnDsv-02 has already been reduced to one-tenth or fewer of the number in the free-living relatives, even though ZnDsv-02 is still widely exposed to the exterior environment. A considerable loss of signal transduction-related genes (~40% or fewer of free-living relatives) has also been observed in two ectosymbionts belonging to *Bacteroidales*, ‘*Candidatus* Symbiothrix dinenymphae’ [30] and phylotype Barb6XT [72] (Fig. S7). Thus, the loss of regulatory genes responsive to environmental changes can occur in ectosymbionts, suggesting that the protist hosts have provided the ectosymbionts with a stable environment that enables such reductive evolution.

However, the overall degree of genome reduction differs markedly between *Desulfovibrio* ectosymbionts and those belonging to *Bacteroidales*. The genome of the former ranges from 1.4 to 1.6 Mb in size, which is approximately half that of their free-living relatives, whereas the latter ranges from 3.6 to 4.8 Mb, which is comparable to their free-living relatives [9, 30, 72]. This difference may be partly attributable to differences in roles necessitated by the symbiotic system. The primary role of the *Desulfovibrio* ectosymbionts appears to be H_2_ oxidation [17, 21], the accumulation of which can suppress fermentation in both protist hosts [73] and co-existing *Endomicrobium* endosymbionts [18, 74]. In contrast, it has been suggested that the *Bacteroidales* ectosymbionts participate in lignocellulose digestion [9, 30, 72], which involves a large number of genes (Supplementary results and Fig. S7). The difference in cell wall structure and device for attachment to the host cell may also contribute to the genome size difference, although genes of ‘unknown function’ (category [S]) accounted for the largest part of the difference (Fig. S7).

It is noteworthy that the ZnDsv-02 population on the single host protist cell was heterogeneous. The frequency of SNPs and indels in ZnDsv-02 was ~20 times higher than in the endosymbionts ‘*Ca*. Adiutrix trichonymphae’ Adiu2019 and ‘*Ca*. Endomicrobium trichonymphae’ Rs-D17 Ti2015 (Table S4). The SNP rate in ZnDsv-02 was also much higher than that in Barb6XT (Table S4) [72]. It is conceivable that genomic variations, probably including rearrangements, led to the failure in reconstructing the complete genome sequence of ZnDsv-02. As the genomes of both ZnDsv-02 and Adiu2019 were reconstructed from the same DNA sample and MiSeq runs, the difference between them would not have been caused by artefacts. It seems unlikely that the sexual reproduction (syngamy) of *Trichonympha* observed in a *Zootermopsis* gut [75] was the cause of ZnDsv-02 genome sequence heterogeneity, as the population of co-existing ‘*Ca*. Adiutrix trichonymphae’ was almost clonal. Thus, we hypothesize that ZnDsv-02 is transmitted horizontally at low frequency among host *T. collaris* cells. As ZnDsv-02 appears to have lost motility, such horizontal transmission would occur only by chance. We expect that ZnDsv-02 cells are typically transmitted vertically.

The presence of genome sequence variants in the ZnDsv-02 population provides an opportunity to infer the process of the reductive genome evolution. Among 1191 genes that contained SNPs/indels, at least 67 genes likely had pseudogenized variants (Dataset S4), and the rate of pseudogenization/absence of orthologous genes in Rs-N31 was much higher (59%) than in the other 1124 genes (16%) (Supplementary results). Furthermore, the NOG profile of the 67 genes was similar to those of pseudogenes of ZnDsv-02 and Rs-N31 (Fig. S4). These suggest that pseudogenization or loss of genes not essential in the symbiotic system is ongoing in the ZnDsv-02 population, and we hypothesize that horizontal transmission might delay the fixation of such slightly deleterious mutations.

In contrast to ZnDsv-02, the frequency of SNPs and indels in the Rs-N31 genome was ~30 times lower than in the two endosymbionts (Table S4). This suggests that those endosymbionts can also undergo chance horizontal transmission, even though the rate would be much lower than that expected for ZnDsv-02. Rs-D17 retains a functional CRISPR-Cas system and several intact RM systems [19, 76], but all CRISPR-Cas and RM systems are pseudogenized in Rs-N31 [21]. This suggests that Rs-D17 can still come into contact with phages that may be phagocytosed with wood particles and other luminal contents by host *Trichonympha* cells, whereas Rs-N31 may not, possibly due to the difference in their specific localization. We therefore hypothesize that cytoplasmic endosymbionts such as Rs-D17 can also be transmitted horizontally among host protist cells via phagocytosis as a very rare occurrence.

An apparently intact CRISPR-Cas system and several RM systems are also present in the ZnDsv-02 genome, suggesting that ZnDsv-02 is exposed to phages and other extracellular DNA. This provides the opportunity for lateral gene transfer (LGT), and indeed, ZnDsv-02 possesses many genes not found in other desulfovibrios (Table S3). Such LGT and phage infection would also contribute to the genomic variations among ZnDsv-02 cells. This situation of ZnDsv-02 with chance horizontal transmission and acquisition of foreign genes is somewhat similar to that of ‘recently host–restricted symbionts’ of insects [77] such as *Serratia symbiotica*, of which genome size is approximately half that of its free-living relatives [78]. However, in ectosymbiosis with protists in the termite gut, host restriction appears to be not enough for massive genome reduction. We suggest that massive genome reduction occurs when bacteria become restricted to a specific function in addition to host restriction, and that genome reduction is initiated even when bacteria can still be transmitted horizontally by chance and acquire genes via LGT. The direct cause of pseudogenization and relatively low G + C content (Fig. S3) in ZnDsv-02 and Rs-N31 could be loss of the *dnaQ* and *fpg* genes, which are required for DNA replication proofreading and repair of oxidized guanine, respectively (Table S7). The loss of *dnaQ* is occasionally observed in intracellular bacteria such as *Mycobacterium leprae* [79] and mycoplasmas [80] and suggested to be the cause of their genome reduction [79, 80]. The loss of *dnaQ* might be advantageous for rapid adaptation to their novel niches.

Interestingly, our 16S rRNA amplicon sequencing analysis detected many ASVs that clustered with the *Desulfovibrio* ectosymbionts of *Trichonympha* in ‘termite cluster 1’ from diverse ‘lower’ termite species that do not harbor *Trichonympha* (Fig. 5b). Our FISH analyses indicated that at least several of those *Desulfovibrio* phylotypes are not associated with any protist cells; they are probably free-swimming bacteria. In any case, it seems likely that the common ancestor of ‘termite cluster 1’ acquired the potential and preference to become an ectosymbiont of protists. A likely key factor would be acquisition of DcuA, which is essential for fumarate respiration [81], via LGT (Fig. S8). DcuA enables the bacteria to proliferate under the low sulfate concentrations in the termite gut [17], if the bacteria can obtain enough malate from the protist host. In turn, *Desulfovibrio* ectosymbionts remove H_2_ in close vicinity to the hydrogenosomes [21].

Although the pattern of gene loss was very similar between ZnDsv-02 and Rs-N31 (Figs. 3 and S6), differences that likely reflect the respective symbiotic relationships were found, in addition to the defense mechanisms. For example, ZnDsv-02 retains most subunits of the type IV secretion system (Fig. S9), which is absent in Rs-N31. As the genome also contains several genes that function in the conjugal transfer system, the type IV secretion system might have played a role in exchanging DNA entities such as plasmids in the ancestor of ZnDsv-02. Because gene components essential for secretion (*virB4*, *B7*) and DNA transfer (*traI*) [71] are psedogenized or absent (Dataset S2 and Table S13), we hypothesize that ZnDsv-02 retains the remaining components only for attachment to the host cell surface, whereas Rs-N31 has completely lost these components. The ability to use lactate is another prominent difference. Utilization of lactate as a carbon and energy source is common in *Desulfovibrio* [82, 83], but the related genes are pseudogenized in Rs-N31. This may indicate that lactate is difficult to take up in the ‘almost intracellular’ lifestyle, and/or that using H_2_ as the sole energy source more benefits the *Trichonympha* host.

The results of our study thus provide new insights into the evolutionary process of cellular symbiosis. Although ZnDsv-02 cells remain widely exposed to the exterior environment as ectosymbionts and can undergo horizontal transmission and LGT events, the genome reduction has already begun. We emphasize the importance of the functional reduction associated with becoming a symbiont, and genome analyses of more diverse mutualistic ectosymbionts may lead to further understanding of mechanisms of genome reduction, which have been investigated mostly in intracellular symbionts and parasitic ectosymbionts [84–86].

Based on differences in 16S rRNA sequence, ANI and AAI [63, 64], as well as its independent acquisition by the host protist lineage, we believe that phylotype ZnDsv-02 should be considered distinct from ‘*Candidatus* Desulfovibrio trichonymphae.’ We therefore propose the novel species name ‘*Candidatus* Desulfovibrio kirbyi’ for this bacterium.

### Description of ‘*Candidatus* Desulfovibrio kirbyi’ sp. nov

Desulfovibrio kirbyi (kir’byi. N.L. gen. n. *Kirbyi*, of Kirby; named after Harold Kirby), a microbiologist who reported the presence of this bacterium as ‘peripheral granules’ in 1932 [87]. The bacteria are short, rod-shaped cells with dimensions of 1.0–2.3 μm (mean ± SD, 1.5 ± 0.3; *n* = 40) by 0.4–0.8 μm (0.6 ± 0.1), based on FISH analysis. The bacteria specifically attach to the cell surface of *T. collaris* in the gut of *Z. nevadensis*. The assignment is based on the 16S rRNA gene sequence (AB444082) and specific hybridization with probe ZnDsv-02-471 (Table S1). This species corresponds to phylotype ZnDsv-02 and the draft genome sequence was deposited in the DDBJ under BioSample SAMD00200503.

## Supplementary information

Supplementary Text

Supplementary Talbes

Supplementary Figures

Dataset S1

Dataset S2

Dataset S3

Dataset S4

Dataset S5

## Data Availability

The sequence data obtained in this study were deposited in the DDBJ under BioProject PRJDB9170 for the genome sequences and the 18S rRNA gene sequences of four *Trichonympha* species. BioProject PRJDB9250 will appear for the 16S rRNA gene sequence datasets of bacteria associated with four *Trichonympha* species. The sequence data for the 16S rRNA ASVs of *Desulfovibrio* obtained from various termite and cockroach species will be available at 10.6084/m9.figshare.12137268.
