# Supplementary material for: Parallel reductive genome evolution in Desulfovibrio ectosymbionts independently acquired by Trichonympha protists in the termite gut
Source: ISME J. 2020 Jun 1;14(9):2288–301. doi: 10.1038/s41396-020-0688-1 (PMC7608387; doi:10.1038/s41396-020-0688-1)
Supplement: Supplementary file 1 — Supplementary Text [file 41396_2020_688_MOESM1_ESM.pdf]

# Supplementary Text

## Supplementary Methods

### Termites and fluorescence in situ hybridization (FISH)

The *Zootermopsis nevadensis* population in Japan was introduced before 1999 from its native habitat in western North America and is considered a hybrid between the two subspecies Z. *nevadensis nevadensis* and *Z. nevadensis nuttingi* [1]. *Reticulitermes speratus* (family Rhinotermitidae) were collected in Saitama Prefecture, Japan, and used for transmission electron microscopy of *Trichonympha agilis*. Before experiments, worker termites were fed with moistened cellulose powder (Nacalai Tesque, Osaka, Japan) for more than 1 week to decrease wood particles in the gut. *Reticulitermes flavipes* were collected in Chattahoochee–Oconee National Forest in Georgia, USA; *Reticulitermes yaeyamanus, Coptotermes formosanus* (Rhinotermitidae), and *Neotermes koshunensis* (family Kalotermitidae) were collected in Okinawa Prefecture, Japan. These termites were kept with their nest logs in the laboratory until being processed for FISH analysis.

FISH analyses for the gut contents of *R. flavipes*, *R. yaeyamanus*, *C. formosanus*, and *N. koshunensis* were conducted as described in the main text, using a combination of probes DSV698 for a broad range of the *Desulfovibrio* genus and EUB338 for most bacteria (Table S1).

### Detection and sequencing of 18S rRNA gene

PCR using a *Eukarya*-specific primer set, E23F3 and E1511R4, for the 18S rRNA gene (Table S2) [2] was conducted to check for the absence or presence of the *Trichonympha* host's genome in WGA products. *TaKaRa Ex Taq* polymerase (Takara Bio, Shiga, Japan) was used for amplification on an S1000 Thermalcycler (Bio-Rad Laboratories, Hercules, CA, USA) under the following conditions: 1 min initial denaturation (1 min at 95°C), 25 cycles of denaturation (15 s at 95°C), annealing (30 s at 58°C), extension (90 s at 72°C) and a final 4 min extension. To phylogenetically identify the host *Trichonympha* species, PCR products from WGA samples of the posterior part or whole cells were purified and subjected to TA cloning and Sanger sequencing as described previously [3].

### Genome sequencing, assembly and binning

The MiSeq raw reads were again trimmed and quality-filtered using platanus_trim v1.0.7 (http://platanus.bio.titech.ac.jp/pltanus_trim) [4]. The paired-end reads were then preliminarily assembled using MEGAHIT v1.3.1 [5], and the mate-pair reads were mapped onto the resulting contigs using Bowtie2 v2.3.4.3. Among the mate-pair reads mapped onto the same location, the read pair with the highest mapping quality was extracted using an in-house program. These MiSeq reads and the PacBio reads were assembled using SPAdes v3.13.1 with the '--only-assembler' option (designated 'contig-set 1'). The PacBio reads alone were also assembled using Canu v1.7 [6] with the '-pacbio-ccs --genomeSize=6m -m 1000' option (contig-set 2) or metaFlye v2.4.2 [7] with the '-pacbio-raw --g 6m' option (contig-set 3). The 55 contigs (see Materials and Methods in the main text) were trimmed into 1 kb and then aligned with these three contig sets using Minimap2 v2.17 [8] with the '-c -p1' option. The top-hit contigs among the contig sets 1–3 were extracted and used for scaffolding with a modified version of Platanus-allee [9] using the contigs as 'long reads'. Gap closing was conducted by aligning other contigs using Minimap2 v2.17 and an in-house program, where possible. After polishing the draft assembly using Pilon v1.22 [10] with four iterations and manual correction, the final contig set was obtained.

## Supplementary Results

### Ratio of sequence reads mapped to genome sequences

Of all of the post-quality filtration reads of MiSeq, 20.7% were mapped onto the ZnDsv-02 draft genome, 34.3% were mapped to the '*Ca.* Adiutrix intracellularis' Adiu2019 genome, 36.9% were mapped to the *Mycoplasmatales* genome, and 0.7% exhibited the highest sequence similarity to the genome of '*Ca.* Endomicrobium trichonymphae' phylotype Rs-D17.

### Analysis of single nucleotide polymorphisms (SNPs) and indels

SNPs and/or indels were found in 1191 of 1257 genes in ZnDsv-02 (Dataset S4). Of these, 147 genes had 'high-impact' variants with SNPs/indels that should cause frameshifts, nonsense mutations and/or loss of start/stop codon. Among the 147 genes, 30 had variants only with indel(s) occurring in homopolymeric regions; these variants might be generated by sequence errors. It was also predicted that 45 genes had 'high-impact' variants with frameshift(s)/nonsense mutation(s) only in the downstream of conserved domains of their deduced protein sequences; these mutations may not critically affect their functions. In addition, 5 genes had variants with an indel in a homopolymeric region and frameshift(s)/nonsense mutation(s) in the downstream of conserved domains. The remaining 67 genes likely have pseudogenized variants (Dataset S4), and these genes do not seem to be essential except for the gene encoding glycyl-tRNA synthetase subunit beta. Indeed, 11 out of the 67 genes are pseudogenized in Rs-N31 and 29 are absent. Among the other 1124 genes with SNPs/indels, 36 are pseudogenized in Rs-N31 and 148 genes are absent in Rs-N31. This difference in frequency of pseudogenization/absence in Rs-N31 between the 67 gene set and the 1124 gene set was statistically significant (one-sided chi-square test, $p < 0.01$). The distribution pattern of these 67 genes among non-supervised orthologous groups (NOGs) shared similarities with that of pseudogenes of ZnDsv-02 (two-sided Spearman's rank correlation test adjusted with Shaffer's modified Bonferroni procedure, $r_s = 0.63$; $p = 0.03$) and Rs-N31 ($r_s = 0.61$; $p = 0.04$), respectively (Fig. S4).

It is noteworthy that the 16S rRNA gene of ZnDsv-02 contained 142 SNPs or indels, which was the largest number among the genes excluding the 67 genes (Dataset S4). Nevertheless, conserved regions corresponding to those of universal PCR primers and FISH probes such as 27F (5'-AGRGTTTGATYMTGGCTCAG-3'), 533F (5'-GCCAGCAGCCGCGGTAA-3'), EUB338 (5'-GCTGCCTCCCGTAGGAGT-3'), 788R (5'-GGACTACHVGGGTATCTAAT-3'), 1390R (5'-ACGGGCGGTGTGTACAA-3') and 1492R (5'-GGHTACCTTGTTACGACTT-3') contained no SNPs or indels, except that there were 2 variants in the mixed-base position of 27F. This suggests that majority of the variants detected in this study were not artefacts generated randomly during sequencing on the MiSeq platform.

### Comparative NOG analysis between *Desulfovibrio* ectosymbionts and *Bacteroidales* ectosymbionts

We compared the total length of genes assigned to each NOG among the *Desulfovibrio* ectosymbionts, *Bacteroidales* ectosymbionts, and their free-living/endosymbiotic relatives (Fig. S7). We analysed the draft genome sequences of '*Candidatus* Symbiothrix dinenymphae' B4-10h (an ectosymbiont of the protist *Dinenympha* sp. from the gut of *R. speratus* [11]), '*Candidatus* Ordinivivax streblomastigis' (an ectosymbiont of the protist *Streblomastix strix* from the gut of *Z. angusticollis* [12]), and Barb6XT (an ectosymbiont of the protist *Barbulanympha* sp. from the gut of the wood-feeding cockroach *Cryptocercus punctulatus* [13]), as well as the complete genome sequences of '*Candidatus* Azobacteroides

pseudotrichonymphae' (an endosymbiont of *Pseudotrichonympha grassii* from the gut of *Coptotermes formosanus*) [14] and their free-living relatives in the order *Bacteroidales*. Except for the category 'unknown function' [S], the greatest difference in the total gene length between the *Desulfovibrio* and *Bacteroidales* ectosymbionts were found in category [G], including glycoside hydrolases involved in lignocellulose digestion; [P], including TonB-dependent SusC/D systems, which transport oligosaccharides; [M], including various glycosyltransferases involved in cell wall formation; and [N], including gliding motility-related genes probably involved in attachment to the host cell [11]. The differences in the total gene length between the *Desulfovibrio* and *Bacteroidales* ectosymbionts were 81,903–532,173 bp in category [G], 125,328–296,136 bp in [P], 108,609–238,377 bp in [M] and 52,347–121,593 bp in [N].

**Acquisition of ectosymbiotic *Desulfovibrio* by *Trichonympha* lineages inferred by phylogenetic analysis**

The phylogeny of *Desulfovibrio* phylotypes exhibited obvious incongruence with that of *Trichonympha* (Fig. 5a). *Desulfovibrio* phylotype ImrTc15 is associated with *Trichonympha globulosa/paraspiralis* (corresponding to ImrTcA/B in Fig. 5a) in *Trichonympha* cluster II [15], which is phylogenetically distinct from cluster I, to which *T. agilis* EH11 and *T. collaris*, hosts for Rs-N31 and ZnDsv-02, belong. Thus, it is safe to say that ImrTc15 was independently acquired. In addition, supposing that the symbiotic association between *Desulfovibrio* and *Trichonympha* cluster I has evolved only once in an ancestral pair, one would have to assume that *Desulfovibrio* ectosymbionts of *Trichonympha* were horizontally transferred to termites that harbour no *Trichonympha* protists, including *Archotermopsis*, *Coptotermes* and *Neotermes*, and that the ectosymbionts have returned back to a free-living life style in those termites. Furthermore, one would need to assume that *Desulfovibrio* ectosymbionts have been lost independently in each of three *Trichonympha* species (*T. sphaerica*, *T. postcylindrica* and *T. campanula*) in *Zootermopsis* termites. We therefore consider that the *Desulfovibrio* ectosymbionts were acquired by *Trichonympha* hosts independently at least three times (Fig. 5a).

**References for Supplementary Methods, Results and Figures**


1. Yashiro T, Mitaka Y, Nozaki T, Matsuura K. Chemical and molecular identification of the invasive termite *Zootermopsis nevadensis* (Isoptera: Archotermopsidae) in Japan. Appl Entomol Zool. 2018; 53: 215–221.
2. Sato T, Kuwahara H, Fujita K, Noda S, Kihara K, Yamada A, et al. Intranuclear verrucomicrobial symbionts and evidence of lateral gene transfer to the host protist in the termite gut. ISME J. 2014; 8: 1008–1019.
3. Murakami T, Segawa T, Bodington D, Dial R, Takeuchi N, Kohshima S, et al. Census of bacterial microbiota associated with the glacier ice worm *Mesenchytraeus solifugus*. FEMS Microbiol Ecol. 2015; 91: fiv003.
4. Kajitani R, Toshimoto K, Noguchi H, Toyoda A, Ogura Y, Okuno M, et al. Efficient de novo assembly of highly heterozygous genomes from whole-genome shotgun short reads. Genome Res. 2014; 24: 1384–1395.
5. Li D, Liu CM, Luo R, Sadakane K, Lam TW. MEGAHIT: an ultra-fast single-node solution for large and complex metagenomics assembly via succinct *de Bruijn* graph. Bioinformatics. 2015; 31: 1674–1676.

6. Koren S, Walenz BP, Berlin K, Miller JR, Bergman NH, Phillippy AM. Canu: scalable and accurate long-read assembly via adaptive *k*-mer weighting and repeat separation. Genome Res. 2017; 27: 722–736.
7. Kolmogorov M, Rayko M, Yuan J, Polevikov E, Pevzner P. metaFlye: scalable long-read metagenome assembly using repeat graphs. bioRxiv. 2019. https://doi.org/10.1101/637637
8. Li H. Minimap2: pairwise alignment for nucleotide sequences. Bioinformatics. 2018; 34: 3094–3100.
9. Kajitani R, Yoshimura D, Okuno M, Minakuchi Y, Kagoshima H, Fujiyama A, et al. Platanus-allee is a de novo haplotype assembler enabling a comprehensive access to divergent heterozygous regions. Nat Commun. 2019; 10: 1702.
10. Walker BJ, Abeel T, Shea T, Priest M, Abouelliel A, Sakthikumar S, et al. Pilon: an integrated tool for comprehensive microbial variant detection and genome assembly improvement. PLoS One. 2014; 9: e112963.
11. Yuki M, Kuwahara H, Shintani M, Izawa K, Sato T, Starns D, et al. Dominant ectosymbiotic bacteria of cellulolytic protists in the termite gut also have the potential to digest lignocellulose. Environ Microbiol. 2015; 17: 4942–4953.
12. Treitli SC, Kolisko M, Husník F, Keeling PJ, Hampl V. Revealing the metabolic capacity of *Streblomastix strix* and its bacterial symbionts using single-cell metagenomics. Proc Natl Acad Sci USA. 2019; 116: 19675–119684.
13. Tai V, Carpenter KJ, Weber PK, Nalepa CA, Perlman SJ, Keeling PJ. Genome evolution and nitrogen fixation in bacterial ectosymbionts of a protist inhabiting wood-feeding cockroaches. Appl Environ Microbiol. 2016; 82: 4682–4695.
14. Hongoh Y, Sharma VK, Prakash T, Noda S, Toh H, Taylor TD, et al. Genome of an endosymbiont coupling $N_2$ fixation to cellulolysis within protist cells in termite gut. Science. 2008; 322: 1108–1109.
15. Ikeda-Ohtsubo W, Brune A. Cospeciation of termite gut flagellates and their bacterial endosymbionts: *Trichonympha* species and '*Candidatus* Endomicrobium trichonymphae'. Mol Ecol. 2009; 18: 332–342.
16. Wallden K, Rivera-Calzada A, Waksman G. Type IV secretion systems: versatility and diversity in function. Cell Microbiol. 2010; 12: 1203–1212.
