# Supplementary material for: Parallel reductive genome evolution in Desulfovibrio ectosymbionts independently acquired by Trichonympha protists in the termite gut
Source: ISME J. 2020 Jun 1;14(9):2288–301. doi: 10.1038/s41396-020-0688-1 (PMC7608387; doi:10.1038/s41396-020-0688-1)
Supplement: Supplementary file 2 — Supplementary Talbes [file 41396_2020_688_MOESM2_ESM.pdf]

Table S1. Oligonucleotide probes for FISH analyses targeting 16S rRNA.

target bacteria	probes or helpers	sequence (5′→3′)	hybridizing temperature (°C)	reference
most groups of bacteria	EUB338	GCTGCCTCCCGTAGGAGT	60	[1]
Desulfovibrio phylotype ZnDsv-02	ZnDsv-02-471	CTGATTAGCACAAGCTGC	51	[2]
	h-ZnDsv-02-436 (helper)	TTTTTTCCCCTCTGACAG	51	This study
	h-ZnDsv-02-472 (helper)	GGTACCATCAACAGCCCG	51	This study
Desulfovibrio	DSV698	GTTCCTCCAGATATCTACGG	51	[3]
'*Ca* . Endomicrobium trichonymphae'	TG1End1023T1	GCTGACTCCCTTGCGGGTCA	51	[4]
'*Ca* . Adiutrix intracellularis'	Delta-ZnvTr3	CTTGAACCGAAGTTCCTG	51	[5]

Table S2. PCR primers used in this study.

target	primer	sequence (5′→3′)	annealing temperature (°C)	reference
16S rRNA gene (*Bacteria*)	341F	CCTACGGGNGGCWGCAG	50	[6]
	785R	GACTACHVGGGTATCTAATCC		
18S rRNA gene (*Eukarya*)	E23F3	ACYTGGTTGATYCTGCC	58	[7]
	E1511R4	CWDCBGCAGGTTCWCCWAC		

Table S3. Genes with higher sequence identity with those of bacteria other than *Desulfovibrio* and its related genera *Lawsonia* and *Bilophila*.

gene ID* **	gene function	gene name of BLAST top hit	organism of BLAST top hit	amino acid sequence identity (%)
ZNDK_0011	Ni2+ ABC transporter permease NikM	KUK13813.1 Cobalamin (Vitamin B12) biosynthesis CbiM protein	bacterium 42_11	39
ZNDK_0020	type II DNA DNA restriction-modification system methylase subunit	KER10083.1 restriction endonuclease subunit M	Candidatus Thermochlorobacteriaceae bacterium GBChlB	74
ZNDK_0047	DUF401 domain-containing protein	SBW09681.1 conserved membrane hypothetical protein	uncultured delta proteobacterium	46
ZNDK_0063	hypothetical protein	WP_061657157.1 platelet activating factor	Bacillus cereus	37
ZNDK_0075	6-pyruvoyltetrahydropterin/6-carboxytetrahydropterin synthase	WP_046500049.1 6-carboxytetrahydropterin synthase QueD	Syntrophomonas zehnderi	37
ZNDK_0076	7-carboxy-7-deazaguanine synthase	WP_026689742.1 radical SAM protein	Bacillus aurantiacus	35
ZNDK_0125	4Fe-4S ferredoxin	SBV90544.1 4Fe-4S ferredoxin iron-sulfur binding domain protein	uncultured delta proteobacterium	56
ZNDK_0126	conserved hypothetical protein	SBW04815.1 conserved exported hypothetical protein	uncultured delta proteobacterium	67
ZNDK_0141	molybdenum cofactor biosynthesis protein MoaA	SBV96375.1 Cyclic pyranopterin monophosphate synthase	uncultured delta proteobacterium	55
ZNDK_0203	DUF3644 domain-containing protein	WP_058437598.1 DUF3644 domain-containing protein	Dehalogenimonas alkenigignens	56
ZNDK_0204	type III restriction-modification system subunit R	WP_090829022.1 type I restriction endonuclease subunit R	Nitrosovibrio tenuis	60
ZNDK_0205	type III restriction-modification system subunit S	PKN93318.1 restriction endonuclease subunit S	Chloroflexi bacterium HGW-Chloroflexi-6	47
ZNDK_0206	type III restriction-modification system subunit M	WP_090627418.1 type I restriction-modification system subunit M	Nitrosomonas marina	70
ZNDK_0214	conserved hypothetical protein	WP_073037206.1 NimC/NimA family protein	Desulfacinum infernum	65
ZNDK_0219	hypothetical protein	OJU33974.1 hypothetical protein BGN99_28150	Alphaproteobacteria bacterium 65-37	38
ZNDK_0235	hypothetical protein	WP_089562420.1 MULTISPECIES: hypothetical protein	Pantoea	40
ZNDK_0281	ATP-dependent helicase	WP_107783531.1 ATP-dependent helicase	Nitrosomonas nitrosa	46
ZNDK_0282	type III restriction-modification system subunit M	RGM83087.1 site-specific DNA-methyltransferase	Bacteroides vulgatus	62
ZNDK_0283	type III restriction-modification system subunit S	WP_055098928.1 restriction endonuclease subunit R	Alcanivorax dieselolei	67
ZNDK_0302	HrgA protein	OLE07885.1 HrgA protein	Delftia sp. 13_1_20CM_4_67_18	71
ZNDK_0315	phosphoadenosine phosphosulfate reductase	WP_015261291.1 PAPS reductase/FAD synthetase	Desulfitobacterium dichloroeliminans	89
ZNDK_0316	hypothetical protein	WP_047830473.1 hypothetical protein	Peptococcaceae bacterium CEB3	56
ZNDK_0345	Cd(II)/Pb(II)-responsive transcriptional regulator	WP_020876686.1 Cd(II)/Pb(II)-responsive transcriptional regulator	Desulfococcus multivorans	70
ZNDK_0346	heavy metal translocating P-type ATPase	KES10271.1 Cation transport ATPase	Snodgrassella alvi SCGC AB-598-O11	75
ZNDK_0354	DNA methyltransferase	AUC61382.1 site-specific DNA-methyltransferase (adenine-specific)	Cyanobacterium sp. HL-69	61
ZNDK_0375	transcriptional regulator	WP_050757692.1 transcriptional regulator	Oxalobacter formigenes	77
ZNDK_0376	copper-exporting ATPase	SBW08937.1 Copper-transporting ATPase 1	uncultured delta proteobacterium	71
ZNDK_0378	hypothetical protein	WP_077999876.1 hypothetical protein	Edwardsiella tarda	48
ZNDK_0379	hypothetical protein	BAF60678.1 hypothetical protein PTH_2497	Pelotomaculum thermopropionicum SI	28
ZNDK_0380	type III restriction-modification system subunit M	WP_090235034.1 site-specific DNA-methyltransferase	Lentibacillus halodurans	66
ZNDK_0456	aspartate aminotransferase	OGP08736.1 proline--tRNA ligase	Deltaproteobacteria bacterium GWA2_45_12	57
ZNDK_0457	50S ribosomal protein L11 methyltransferase	WP_045670498.1 pyridoxal phosphate-dependent aminotransferase	Paenibacillus beijingensis	51
ZNDK_0470	TRAP transporter large permease	WP_066605601.1 HAD-IIA family hydrolase	Desulfomicrobium orale	65
ZNDK_0471	TRAP transporter small permease	WP_066605604.1 TRAP transporter large permease	Desulfomicrobium orale	83
ZNDK_0472	TRAP transporter substrate-binding protein	SBV90749.1 conserved membrane hypothetical protein	uncultured delta proteobacterium	67
ZNDK_0473	anaerobic glycerol-3-phosphate dehydrogenase subunit A	WP_066605612.1 TRAP transporter substrate-binding protein	Desulfomicrobium orale	81
ZNDK_0474	anaerobic glycerol-3-phosphate dehydrogenase subunit B	WP_066605614.1 anaerobic glycerol-3-phosphate dehydrogenase subunit A	Desulfomicrobium orale	71
ZNDK_0475	anaerobic glycerol-3-phosphate dehydrogenase subunit C	WP_083517935.1 anaerobic glycerol-3-phosphate dehydrogenase subunit B	Desulfomicrobium orale	56
ZNDK_0476	DNA-directed RNA polymerase subunit beta	WP_066608825.1 anaerobic glycerol-3-phosphate dehydrogenase subunit C	Desulfomicrobium orale	61
ZNDK_0486	type III restriction-modification system subunit M	BBD76785.1 cell filamentation protein Fic	Hydrogenophilus thermoluteolus	59
ZNDK_0505	anaerobic ribonucleoside-triphosphate reductase activating protein	SBW05443.1 NrdD	uncultured delta proteobacterium	73
ZNDK_0506	MOSC domain-containing molybdenum cofactor biosynthesis protein	WP_084068205.1 anaerobic ribonucleoside-triphosphate reductase activating protein	Desulfobacterium vacuolatum	47
ZNDK_0520	DUF1819 domain-containing protein	WP_041220246.1 BrxE family protein	Dehalogenimonas lykanthroporepellens	49
ZNDK_0521	DUF1788 domain-containing protein	WP_013219026.1 DUF1819 family protein	Dehalogenimonas lykanthroporepellens	65
ZNDK_0522	BREX system P-loop protein	ADJ26905.1 hypothetical protein Dehly_1627	Dehalogenimonas lykanthroporepellens BL-DC-9	76
ZNDK_0523	BREX-1 system adenine-specific DNA-methyltransferase	WP_041220579.1 BREX system P-loop protein BrxC	Dehalogenimonas lykanthroporepellens	73
ZNDK_0524	ATP-binding protein	ADJ26903.1 conserved hypothetical protein	Dehalogenimonas lykanthroporepellens BL-DC-9	66
ZNDK_0526	alkaline phosphatase	PID44693.1 hypothetical protein CSB47_10860	Proteobacteria bacterium	57
ZNDK_0527	BREX system Lon protease-like protein	WP_013219022.1 PglZ domain-containing protein	Dehalogenimonas lykanthroporepellens	67
ZNDK_0528	hypothetical protein	WP_013219021.1 BREX system Lon protease-like protein BrxL	Dehalogenimonas lykanthroporepellens	79
ZNDK_0543	Fe(3+) ions import ATP-binding protein	SBV97846.1 ABC transporter, permease protein	uncultured delta proteobacterium	86
ZNDK_0544	multidrug transporter subunit MdtC	SBV97839.1 Spermidine/putrescine import ATP-binding protein PotA	uncultured delta proteobacterium	73
ZNDK_0569	cation diffusion facilitator family transporter	KMY68636.1 hypothetical protein AAU61_03115	Desulfocarbo indianensis	55
ZNDK_0570	MarR family transcriptional regulator	WP_092752876.1 cation transporter	Hydrogenoanaerobacterium saccharovorans	39
ZNDK_0573	acetate kinase	WP_085834031.1 O-acetylhomoserine aminocarboxypropyltransferase/cysteine synthase	Clostridium merdae	75
ZNDK_0580	fumarate reductase subunit C	WP_012987134.1 fumarate reductase subunit FrdD	Xenorhabdus bovienii	55
ZNDK_0581	fumarate reductase subunit B	WP_105571186.1 fumarate reductase subunit FrdC	Cronobacter sakazakii	60
ZNDK_0582	fumarate reductase flavoprotein subunit A	WP_032906821.1 MULTISPECIES: succinate dehydrogenase/fumarate reductase iron-sulfur subunit	Yersinia	73
ZNDK_0583	LysR family transcriptional regulator	WP_095846832.1 fumarate reductase (quinol) flavoprotein subunit	Gibbsiella quercinecans	78

ZNDK_0584	fumarate hydratase, alpha subunit	WP_073591285.1 LysR family transcriptional regulator	Anaerocolumna xylanovorans	33
ZNDK_0601	uncharacterized transcriptional regulator	PYJ56808.1 iron-sulfur cluster loop	Verrucomicrobia bacterium	34
ZNDK_0602	hypothetical protein	SBW04119.1 Uncharacterized HTH-type transcriptional regulator in smaI restriction system 5'region (modular protein)	uncultured delta proteobacterium	76
ZNDK_0603	uncharacterized transcriptional regulator	WP_068066448.1 hypothetical protein	Rheinheimera sp. SA_1	35
ZNDK_0604	conserved hypothetical protein	SBW04119.1 Uncharacterized HTH-type transcriptional regulator in smaI restriction system 5'region (modular protein)	uncultured delta proteobacterium	73
ZNDK_0616	type I restriction-modification system subunit S	WP_088891394.1 type I restriction-modification system subunit M	Leptolyngbya ohadii	80
ZNDK_0617	type I restriction-modification system subunit R	PCI76318.1 restriction endonuclease	Ectothiorhodospiraceae bacterium	67
ZNDK_0618	primosomal protein N'	WP_088891389.1 type I restriction endonuclease subunit R	Leptolyngbya ohadii	80
ZNDK_0631	RNA polymerase subunit sigma-24	WP_095097827.1 hypothetical protein	Enterobacter sp. 10-1	38
ZNDK_0632	Zn-dependent M50 family peptidase	WP_084880213.1 sigma-70 family RNA polymerase sigma factor	Pantoea cypripedii	51
ZNDK_0661	hypothetical protein	PKN07440.1 SAM-dependent methyltransferase	Deltaproteobacteria bacterium HGW-Deltaproteobacteria-8	62
ZNDK_0662	hypothetical protein	WP_080700917.1 hypothetical protein	Bordetella hinzii	56
ZNDK_0663	glutamine-dependent NAD+ synthetase	WP_080700918.1 ATP-binding protein	Bordetella hinzii	72
ZNDK_0665	ArsR family transcriptional regulator	SBV93787.1 Methyltransferase domain-containing protein (fragment)	uncultured delta proteobacterium	75
ZNDK_0672	conserved hypothetical protein	WP_041686707.1 Zn-dependent hydrolase	Ethanoligenens harbinense	61
ZNDK_0694	CRISPR-associated protein Cas1 / type I-C	WP_005876723.1 CRISPR-associated endonuclease Cas2	Oxalobacter formigenes	72
ZNDK_0696	CRISPR-associated protein Cas3 / type I	WP_005876726.1 CRISPR-associated protein Cas4	Oxalobacter formigenes	54
ZNDK_0697	CRISPR-associated protein Cas7 / type I-C	OFY32152.1 CRISPR-associated protein	Bacteroidetes bacterium GWF2_43_11	44
ZNDK_0698	hypothetical protein	OGS25054.1 CRISPR-associated protein	Elusimicrobia bacterium RIFOXYB2_FULL_50_12	65
ZNDK_0699	CRISPR-associated protein Cas5 / type I	OGS25053.1 hypothetical protein A2314_01970	Elusimicrobia bacterium RIFOXYB2_FULL_50_12	34
ZNDK_0700	Xaa-Pro dipeptidase	PKN20006.1 CRISPR-associated protein Cas5	Deltaproteobacteria bacterium HGW-Deltaproteobacteria-6	62
ZNDK_0702	putative dihydropyrimidinase	WP_039382942.1 MULTISPECIES: shikimate kinase AroL	Pantoea	52
ZNDK_0711	H+-translocating [NiFe] hydrogenase complex, iron-sulfur subunit CooF	SBV91239.1 Sodium/alanine symporter AgcS	uncultured delta proteobacterium	61
ZNDK_0737	putative exoribonuclease II	PIX65152.1 hypothetical protein COZ43_10165	Sphingomonadales bacterium CG_4_10_14_3_um_filter_58_15	55
ZNDK_0741	DNA adenine methylase	WP_062376142.1 hypothetical protein	Treponema endosymbiont of Eucomonympha sp.	61
ZNDK_0742	tRNA threonylcarbamoyladenosine biosynthesis protein tsaC	WP_015713118.1 DNA adenine methylase	Treponema azotonutricium	64
ZNDK_0750	protein HflK	WP_038351313.1 sodium:solute symporter	Eubacterium limosum	72
ZNDK_0784	30S ribosomal protein S20	SBV90540.1 conserved exported hypothetical protein	uncultured delta proteobacterium	50
ZNDK_0794	glutamyl-tRNA synthetase	WP_077533345.1 methylated-DNA--	protein	68
ZNDK_0803	conserved hypothetial protein	OGS37623.1 thiamine biosynthesis protein ThiS	Elusimicrobia bacterium RIFOXYB2_FULL_49_7	56
ZNDK_0818	conserved hypothetical protein	WP_013446319.1 7-cyano-7-deazaguanine synthase QueC	Paludibacter propionicigenes	46
ZNDK_0842	hypothetical protein	SBW05763.1 conserved hypothetical protein	uncultured delta proteobacterium	67
ZNDK_0843	DNA-cytosine methyltransferase	WP_096900009.1 hypothetical protein	Klebsiella pneumoniae	61
ZNDK_0844	adenine-specific DNA-methyltransferase	WP_109552866.1 DNA cytosine methyltransferase	Escherichia coli	65
ZNDK_0845	hypothetical protein	WP_006682463.1 adenine-specific DNA-methyltransferase	Candidatus Glomeribacter gigasporarum	70
ZNDK_0846	glutamine-dependent NAD+ synthetase	WP_006682462.1 hypothetical protein	Candidatus Glomeribacter gigasporarum	76
ZNDK_0870	conserved hypothetical protein	OGV55443.1 cystathionine gamma-synthase	Lentisphaerae bacterium GWF2_52_8	59
ZNDK_0883	High-molecular-weight cytochrome c subunit D	KPK25183.1 hypothetical protein AMJ61_12665	Desulfobacterales bacterium SG8_35_2	48
ZNDK_0897	helicase	SYW88659.1 RNA helicase	Candidatus Methylomirabilis sp. lanth	50
ZNDK_0898	hypothetical protein	AEG16695.1 protein of unknown function DUF1156	Desulfofundulus kuznetsovii DSM 6115	54
ZNDK_0899	hypothetical protein	SYW88653.1 hypothetical protein MELA_01056	Candidatus Methylomirabilis sp. lanth	52
ZNDK_0900	nucleotidyltransferase	WP_050865940.1 SLATT domain-containing protein	Burkholderia pseudomallei	67
ZNDK_0901	hypothetical protein	WP_088958127.1 nucleotidyltransferase	Variovorax sp. HW608	64
ZNDK_0902	adenylyltransferase/sulfurtransferase MoeZ	WP_062359346.1 hypothetical protein	Halomonas axialensis	52
ZNDK_0903	molybdopterin synthase sulfur carrier subunit	AEF86955.1 Uba/ThiF-type NAD/FAD-binding protein	Treponema primitia ZAS-2	71
ZNDK_0904	M67 family peptidase	WP_039324934.1 MoaD/ThiS family protein	Pectobacterium betavasculorum	58
ZNDK_0905	cysteine synthase	WP_083961043.1 MULTISPECIES: M67 family peptidase	Dickeya	60
ZNDK_0906	phosphoglycerate mutase	WP_095845737.1 cysteine synthase family protein	Gibbsiella quercinecans	68
ZNDK_0909	nitronate monooxygenase	WP_027320336.1 aspartate ammonia-lyase	Bacillus sp. URHB0009	69
ZNDK_0926	Alw26I/Eco31I/Esp3I family type II restriction-modification system subunit R	WP_081783039.1 Alw26I/Eco31I/Esp3I family type II restriction adenine-specific DNA-methyltransferase	Lachnospiraceae bacterium AC3007	52
ZNDK_0927	multidrug efflux pump priplasmic subunit AcrA	AAQ72363.1 BsmAI endonuclease	Geobacillus stearothermophilus	55
ZNDK_0932	RNA polymerase-binding protein DksA	SBV95429.1 putative Diguanylate cyclase	uncultured delta proteobacterium	52
ZNDK_0934	ATP-dependent protease ATPase/chaperone subunit HslU	WP_092752876.1 cation transporter	Hydrogenoanaerobacterium saccharovorans	42
ZNDK_0944	diaminopropionate ammonia-lyase YgeX	SBV96853.1 conserved hypothetical protein	uncultured delta proteobacterium	65
ZNDK_0949	uncharacterized amidohydrolase SsnA	SBV96844.1 D-stereospecific phenylhydantoinase	uncultured delta proteobacterium	58
ZNDK_0950	putative selenate reductase	SBV96838.1 Amidohydrolase	uncultured delta proteobacterium	68
ZNDK_0951	putative nucleobase : H+ symporter	SBV96833.1 hypothetical protein KL86DPRO_11112	uncultured delta proteobacterium	55
ZNDK_0955	nitrate/sulfonate/bicarbonate ABC transporter substrate-binding protein	OTE98016.1 hypothetical protein BCS42_11305	Crenothrix sp. D3	51
ZNDK_0956	ABC transporter permease	OGB03577.1 ABC transporter substrate-binding protein	Burkholderiales bacterium RIFCSPHIGHO2_12_FULL_63_20	65
ZNDK_0957	uncharacterized selenium metabolism protein YedF	WP_037982867.1 ABC transporter permease	Thauera sp. 27	56
ZNDK_0958	selenophosphate synthase	SBV96798.1 conserved hypothetical protein	uncultured delta proteobacterium	60
ZNDK_0959	molybdenum cofactor cytidylyltransferase	SBV96792.1 Selenide, water dikinase	uncultured delta proteobacterium	67
ZNDK_0960	YqeC-like putative molybdenum hydroxylase maturation protein	SBV96858.1 conserved hypothetical protein	uncultured delta proteobacterium	53

ZNDK_0961	selenium-dependent molybdenum hydroxylase accessory protein YqeB	SBV96865.1 conserved hypothetical protein	uncultured delta proteobacterium	53
ZNDK_1009	cation efflux system protein CusC	WP_043015501.1 MULTISPECIES: copper-binding protein	Citrobacter	49
ZNDK_1042	putative Fe2+ permease periplasmic component	OCG35112.1 iron permease	Gilliamella apicola	55
ZNDK_1044	ABC efflux transporter permease	WP_015708650.1 DUF2318 domain-containing protein	Treponema primitia	37
ZNDK_1045	ABC efflux transporter permease	WP_101698377.1 ABC transporter permease	Clostridium minihomine	61
ZNDK_1046	ABC efflux transporter ATP-binding protein	WP_065798102.1 ABC transporter permease	Gilliamella apicola	51
ZNDK_1047	cytochrome c class III	WP_073078457.1 ABC transporter ATP-binding protein	Sporobacter termitidis	76
ZNDK_1053	glutamate racemase	OQX25494.1 hypothetical protein BWK80_15215	Desulfobacteraceae bacterium IS3	59
ZNDK_1057	DNA repair/recombination protein RecR	SBV99400.1 conserved hypothetical protein	uncultured delta proteobacterium	63
ZNDK_1105	DNA helicase II	KGP76652.1 hypothetical protein JT05_03450	Desulfosporosinus sp. Tol-M	86
ZNDK_1106	hypothetical protein	KGP76651.1 hypothetical protein JT05_03445	Desulfosporosinus sp. Tol-M	77
ZNDK_1107	helicase domain protein	KGP76650.1 hypothetical protein JT05_03440	Desulfosporosinus sp. Tol-M	64
ZNDK_1108	hypothetical protein	KGP76649.1 hypothetical protein JT05_03435	Desulfosporosinus sp. Tol-M	84
ZNDK_1109	hypothetical protein	KGP76648.1 hypothetical protein JT05_03430	Desulfosporosinus sp. Tol-M	89
ZNDK_1110	radical SAM domain protein	KGP76647.1 hypothetical protein JT05_03425	Desulfosporosinus sp. Tol-M	88
ZNDK_1111	transposase	CCY04073.1 radical SAM domain protein	Faecalibacterium sp. CAG:1138	62
ZNDK_1112	helicase domain protein	BAA17360.1 transposase	Synechocystis sp. PCC 6803	47
ZNDK_1113	putative DNA restriction-modification system endonuclease subunit	WP_018214272.1 DUF3883 domain-containing protein	Desulfitobacterium hafniense	78
ZNDK_1114	DNA restriction-modification system adenine-specific methylase subunit	OGR36794.1 hypothetical protein A2091_01035	Desulfuromonadales bacterium GWD2_61_12	38
ZNDK_1115	phage integrase	WP_015942905.1 DUF1156 domain-containing protein	Desulfitobacterium hafniense	69
ZNDK_1117	hypothetical protein	SBW07532.1 Transmembrane protein 145	uncultured delta proteobacterium	75
ZNDK_1118	hypothetical protein	SBW07526.1 hypothetical protein KL86DPRO_30072	uncultured delta proteobacterium	50
ZNDK_1121	hypothetical protein	SBW00921.1 hypothetical protein KL86DPRO_11866	uncultured delta proteobacterium	62
ZNDK_1122	hypothetical protein	ODT05158.1 hypothetical protein ABS52_00150	Gemmatimonadetes bacterium SCN 70-22	46
ZNDK_1123	hypothetical protein	EGV16088.1 hypothetical protein ThimaDRAFT_4636	Thiocapsa marina 5811	64
ZNDK_1124	hypothetical protein	SBW07526.1 hypothetical protein KL86DPRO_30072	uncultured delta proteobacterium	63
ZNDK_1125	hypothetical protein	SBW07521.1 conserved hypothetical protein	uncultured delta proteobacterium	63
ZNDK_1126	hypothetical protein	SBW00921.1 hypothetical protein KL86DPRO_11866	uncultured delta proteobacterium	67
ZNDK_1127	conjugal transfer protein TraL	SBW07515.1 hypothetical protein KL86DPRO_30070	uncultured delta proteobacterium	49
ZNDK_1131	putative type IV secretion system component virB2	PWM60705.1 hypothetical protein DBX91_04335	Subdoligranulum variabile	61
ZNDK_1136	putative type IV secretion system component virB9	SBW08311.1 Conjugal transfer protein	uncultured delta proteobacterium	81
ZNDK_1139	type II toxin-antitoxin system HicB family antitoxin	SBW08319.1 putative conjugal transfer protein TrbI	uncultured delta proteobacterium	74
ZNDK_1140	type II toxin-antitoxin system HicB family antitoxin	WP_066608088.1 type II toxin-antitoxin system HicB family antitoxin	Desulfomicrobium orale	38
ZNDK_1143	putaive type IV secretion system, signal peptidase TraF	SBW00997.1 Conjugal transfer protein TraG	uncultured delta proteobacterium	80
ZNDK_1148	GTPase EngA	SBW01128.1 conserved hypothetical protein	uncultured delta proteobacterium	67
ZNDK_1150	hypothetical protein	RFT07290.1 type I restriction endonuclease subunit R	Ruminococcaceae bacterium KLE1738	70
ZNDK_1151	type I restriction-modification system subunit S	WP_056253481.1 hypothetical protein	Flavobacterium sp. Leaf82	56
ZNDK_1153	hypothetical protein	PWM00978.1 restriction endonuclease subunit M	Selenomonadales bacterium	81
ZNDK_1154	tRNA-specific 2-thiouridylase MnmA	WP_038324540.1 ATP-dependent sacrificial sulfur transferase LarE	bacterium MS4	64
ZNDK_1155	adenylyltransferase/sulfurtransferase MoeZ	CCZ21584.1 tRNA-specific 2-thiouridylase MnmA	Acetobacter sp. CAG:977	52
ZNDK_1157	conserved hypothetical protein	PMP72858.1 MFS transporter	Calditerrivibrio nitroreducens	34
ZNDK_1195	lytic transglycosylase	WP_074009390.1 hypothetical protein	Duodenibacillus massiliensis	49
ZNDK_1206	conjugal transfer system, relaxosome component TraJ	SBW01094.1 conserved exported hypothetical protein	uncultured delta proteobacterium	60
ZNDK_1209	4-carboxymuconolactone decarboxylase	SBV93187.1 hypothetical protein KL86DPRO_10490	uncultured delta proteobacterium	50
ZNDK_1210	4-carboxymuconolactone decarboxylase	WP_101250335.1 4-carboxymuconolactone decarboxylase	Telmatospirillum siberiense	64
ZNDK_1211	flavodoxin	WP_045667209.1 cupin domain-containing protein	Geobacter sulfurreducens	64
ZNDK_1216	DNA restriction-modification system endonuclease subunit	WP_002234403.1 MULTISPECIES: site-specific DNA-methyltransferase	Neisseria	71
ZNDK_1217	multidrug efflux pump subunit MdtB	WP_081642720.1 MvaI/BcnI restriction endonuclease family protein	Aquaspirillum serpens	66
ZNDK_1224	BCCT transporter	WP_077072100.1 phosphodiesterase	Mailhella massiliensis	74
ZNDK_1229	periplasmic sirohydrochlorin cobaltochelatase/ferrochelatase	OGM20735.1 hypothetical protein A2714_03580	Candidatus Woesebacteria bacterium RIFCSPHIGHO2_01_FULL_38_9	34
ZNDK_1236	transposase	WP_101088568.1 anaerobic C4-dicarboxylate transporter	Shewanella sp. ALD9	69
ZNDK_1238	multiple promoter invertase	SFM14751.1 hypothetical protein SAMN05421830_11669	Desulfomicrobium norvegicum	71
ZNDK_1239	hydrogenase maturation protease	WP_101911337.1 invertase	Marasmitruncus massiliensis	82
ZNDK_1241	ammonia-forming cytochrome c nitrite reductase large subunit	ABB27653.1 DEAD/DEAH box helicase-like protein	Chlorobium chlorochromatii CaD3	67

* Red denotes the genes also found in 'Ca. Desulfovibrio trichonymphae' phylotype Rs-N31.

** Orange shade denotes the genes possibly obtained via lateral gene transfer from other bacteria in the termite gut.

Table S4. Variant (SNP and indel) rate of the genomes of ecto- and endosymbiotic bacteria associated with *Trichonympha* protists.

bacteria*	life style	original average read depth	genome size (Mb)	SNPs (/site)	indels (/site)	total (/site)	reference
Desulfovibrio phylotype ZnDsv-02	ectosymbiont	204	1.6	1/66	1/862	1/61	this study
'*Ca*. Adiutrix intracellularis' genomovar Adiu2019	endosymbiont	257	2.1	1/1,464	1/8,320	1/1,245	this study
'*Ca*. Desulfovibrio trichonymphae' Rs-N31	ectosymbiont ('almost intracellular')	795	1.4	1/235,009	1/44,064	1/37,106	this study
'*Ca.* Endomicrobium trichonymphae' Rs-D17 genomovar Ti2015	endosymbiont	289	1.1	1/2,011	1/3,618	1/1,292	this study
Ectosymbiont Barb6XT of *Barbulanympha* sp.	ectosymbiont	-	3.6	1/10,833**	-	-	[8]

* The genome sequences of ZnDsv-02 and Adiu2019 were obtained from an identical DNA sample and MiSeq run.
Similarly, the genomes of Rs-N31 and Rs-D17 genomovar Ti2015 were obtained from an identical DNA sample and MiSeq run.
** Calculated using the data in the reference study.

Table S5. Frequency of reads with different SNP patterns at two regions of the ZnDsv-02 genome.

contig ID	location in the genome	read phylotypes	number of reads
contig_8	32,680–32,780	type 1	11
		type 2	10
		type 3	10
		type 4	9
		type 5	4
		type 6	3
		type 7	2
		type 8	1
contig_9	46,520–46,620	type 1	11
		type 2	11
		type 3	6
		type 4	6
		type 5	4
		type 6	3

Table S6. Average nucleotide identity (ANI) and average amino acid identity (AAI) between the genomes of *Desulfovibrio* phylotype ZnDsv-02 and its relatives.

Desulfovibrio phylotype ZnDsv-02 vs.	ANI (%)	AAI (%)
'*Ca* . Desulfovibrio trichonymphae' Rs-N31	78	70
D. desulfuricans ATCC 27774	77	67
D. fairfieldensis CCUG 45958	76	69
Desulfovibrio sp. G11	76	66
D. piger FI11049	77	64

Table S7. Presence or absence of genes known to be involved in DNA repair and recombination.

classification	gene	function	*Desulfovibrio* phylotype ZnDsv-02	'*Ca* . Desulfovibrio trichonymphae' Rs-N31	*D. desulfuricans* ATCC 27774	*D. fairfieldensis* CCUG 45958
DNA replication	*dnaB*	replicative DNA helicase	+	+	+	+
	dnaE	DNA polymerase III subunit alpha	+	+	+	+
	dnaG	DNA primase	+	+	+	+
	dnaN	DNA polymerase III subunit beta	+	+	+	+
	dnaQ	DNA polymerase III subunit epsilon	-	-	+	+
	dnaX	DNA polymerase III subunit gamma/tau	+	+	+	+
	holA	DNA polymerase III subunit delta	+	+	+	+
	holB	DNA polymerase III subunit delta'	+	+	+	+
	holC	DNA polymerase III subunit chi	-	-	-	-
	holD	DNA polymerase III subunit psi	-	-	-	-
	holE	DNA polymerase III subunit theta	-	-	-	-
	rnhA	ribonuclease HI	+	+	+	+
	rnhB	ribonuclease HII	+	+	+	+
	rhnC	ribonuclease HIII	-	-	-	-
	ssb	single-strand DNA-binding protein	+	+	+	+
	polA	DNA polymerase I	+	+	+	+
	ligA	NAD-dependent DNA ligase	+	+	+	+
Base excision repair	*alkA*	DNA-3-methyladenine glycosylase II	-	-	+	+
	fpg	formamidopyrimidine-DNA glycosylase	-	-	+	+
	mpg	DNA-3-methyladenine glycosylase	-	-	-	-
	mug	double-stranded uracil-DNA glycosylase	-	-	-	-
	mutY	A/G-specific adenine glycosylase	+	+	+	+
	nei	endonuclease VIII	-	-	-	-
	nfo	deoxyribonuclease IV	-	-	-	-
	nth	endonuclease III	+	+	+	+
	ogg1	N-glycosylase/DNA lyase	-	-	-	-
	smug1	single-strand selective monofunctional uracil DNA glycosylase	-	-	-	-
	tag	DNA-3-methyladenine glycosylase I	+	+	-	-
	udg	uracil-DNA glycosylase	-	-	-	-
	ung	uracil-DNA glycosylase	-	-	-	-
	xthA	exodeoxyribonuclease III	+	+	+	+
Nucleotide excision repair	*mfd*	transcription-repair coupling factor	+	+	+	+
	uvrA	excinuclease ABC subunit A	+	+	+	+
	uvrB	excinuclease ABC subunit B	+	+	+	+
	uvrC	excinuclease ABC subunit C	+	+	+	+
	uvrD	DNA helicase II UvrD	+	+	+	+
Mismatch repair	*dam*	DNA adenine methylase	+	-	-	+
	exoI	exodeoxyribonuclease I	-	-	-	-
	exoX	exodeoxyribonuclease X	-	-	-	-
	mutL	DNA mismatch repair protein MutL	+	+	+	+
	mutH	DNA mismatch repair protein MutH	-	-	-	-
	mutS	DNA mismatch repair protein MutS	+	+	+	+
	xseA	exodeoxyribonuclease VII large subunit	+	+	+	+
	recJ	single-stranded-DNA-specific exonuclease	+	+	+	+
Homologous recombination	*dnaT*	DNA replication protein DnaT	-	-	-	-
	recA	DNA recombinase RecA	+	+	+	+
	recB	exodeoxyribonuclease V beta subunit	-	-	-	-
	recC	exodeoxyribonuclease V gamma subunit	-	-	-	-
	recD	exodeoxyribonuclease V alpha subunit	+	+	+	+
	recF	DNA replication and repair protein RecF	-	-	-	-
	recG	ATP-dependent DNA helicase RecG	-	-	-	-
	recO	DNA repair/recombination protein RecO	+	+	+	+
	recR	DNA repair/recombination protein RecR	+	+	+	+
	ruvA	holliday junction DNA helicase RuvA	+	+	+	+
	ruvB	holliday junction DNA helicase RuvB	+	+	+	+
	ruvC	holliday junction resolvase RuvC	+	+	+	+
	priA	primosomal protein N'	+	+	+	+
	priB	primosomal replication protein N	-	-	-	-
	priC	primosomal replication protein N''	-	-	-	-

Table S8. Pseudogenes of *Desulfovibrio* phylotype ZnDsv-02 and their corresponding non-supervised orhologous groups (NOGs).

#	pseudogene ID	NOG*	Function
1	ZNDK_pg_16	C	pseudogene of FeS-binding protein
2	ZNDK_pg_53	C	pseudogene of bifunctional acetaldehyde-CoA/alcohol dehydrogenase
3	ZNDK_pg_66	C	pseudogene of aldo/keto reductase
4	ZNDK_pg_01	D	pseudogene of flagellar synthesis regulator
5	ZNDK_pg_20	E	pseudogene of acetylglutamate kinase
6	ZNDK_pg_54	E	pseudogene of nitrogen regulatory protein P-II
7	ZNDK_pg_77	E	pseudogene of aspartate ammonia-lyase
8	ZNDK_pg_78	E	pseudogene of aspartate ammonia-lyase
9	ZNDK_pg_14	F	pseudogene of carbamoyl-phosphate synthase large subunit
10	ZNDK_pg_13	G	pseudogene of 1,4-alpha-glucan-branching protein
11	ZNDK_pg_17	G	pseudogene of MFS transporter
12	ZNDK_pg_49	G	pseudogene of sugar kinase
13	ZNDK_pg_02	H	pseudogene of bifunctional adenosylcobinamide kinase/ adenosylcobinamide-phosphate guanylyltransferase
14	ZNDK_pg_39	H	pseudogene of cobalamin synthase
15	ZNDK_pg_41	H	pseudogene of cobyric acid synthase
16	ZNDK_pg_55	H	pseudogene of cobalamin biosynthesis protein CbiG
17	ZNDK_pg_70	H	pseudogene of cobyric acid synthase
18	ZNDK_pg_73	H	pseudogene of cobalamin biosynthesis protein CbiG
19	ZNDK_pg_74	H	pseudogene of molybdenum cofactor biosynthesis protein A
20	ZNDK_pg_75	H	pseudogene of precorrin-6Y C5,15-methyltransferase (decarboxylating)
21	ZNDK_pg_85	H	pseudogene of cobyrinic acid a,c-diamide synthase
22	ZNDK_pg_03	J	pseudogene of glutamyl-Q tRNA(Asp) synthetase
23	ZNDK_pg_30	J	pseudogene of ribosomal subunit interface protein
24	ZNDK_pg_31	J	pseudogene of translation elongation factor G
25	ZNDK_pg_32	J	pseudogene of metallo-beta-lactamase
26	ZNDK_pg_35	K	pseudogene of ROS/MucR family transcriptional regulator
27	ZNDK_pg_37	K	pseudogene of RNA polymerase subunit sigma-70
28	ZNDK_pg_04	L	pseudogene of integrase
29	ZNDK_pg_08	L	pseudogene of endonuclease
30	ZNDK_pg_11	L	pseudogene of transposase
31	ZNDK_pg_12	L	pseudogene of ATP-dependent endonuclease
32	ZNDK_pg_24	L	pseudogene of transcriptional regulator
33	ZNDK_pg_28	L	pseudogene of transposase

34	ZNDK_pg_34	L	pseudogene of integrase
35	ZNDK_pg_40	L	pseudogene of transposase
36	ZNDK_pg_43	L	pseudogene of integrase
37	ZNDK_pg_44	L	pseudogene of transposase
38	ZNDK_pg_50	L	pseudogene of transposase
39	ZNDK_pg_52	L	pseudogene of transposase
40	ZNDK_pg_61	L	pseudogene of putative antirestrictionl protein ArdC
41	ZNDK_pg_67	L	pseudogene of transposase
42	ZNDK_pg_68	L	pseudogene of transposase
43	ZNDK_pg_69	L	pseudogene of helicase
44	ZNDK_pg_29	M	pseudogene of polysaccharide export protein
45	ZNDK_pg_36	M	pseudogene of VIT-vWA domains-containing uncharacterized protein
46	ZNDK_pg_72	M	pseudogene of outermembrane Hek-like protein
47	ZNDK_pg_06	N	pseudogene of flagellar motor switch protein
48	ZNDK_pg_48	N	pseudogene of flagellar motor switch protein
49	ZNDK_pg_82	O	pseudogene of U32 family peptidase
50	ZNDK_pg_23	P	pseudogene of Fe3+-complex ABC transporter permease
51	ZNDK_pg_51	P	pseudogene of ammonia-forming cytochrome c nitrite reductase large subunit
52	ZNDK_pg_86	P	pseudogene of low-affinity inorganic phosphate transporter
53	ZNDK_pg_18	Q	pseudogene of phenylacetate--CoA ligase
54	ZNDK_pg_05	S	pseudogene of hypothetical protein
55	ZNDK_pg_07	S	pseudogene of hypothetical protein
56	ZNDK_pg_09	S	pseudogene of uncharacterized metal-dependent phosphoesterase
57	ZNDK_pg_15	S	pseudogene of MBL fold metallo-hydrolase
58	ZNDK_pg_19	S	pseudogene of hypothetical protein
59	ZNDK_pg_21	S	pseudogene of hypothetical protein
60	ZNDK_pg_22	S	pseudogene of conserved hypothetical protein
61	ZNDK_pg_26	S	pseudogene of cell division protein Fic
62	ZNDK_pg_33	S	pseudogene of hypothetical protein
63	ZNDK_pg_38	S	pseudogene of hypothetical protein
64	ZNDK_pg_42	S	pseudogene of hypothetical protein
65	ZNDK_pg_45	S	pseudogene of hypothetical protein
66	ZNDK_pg_46	S	pseudogene of conserved hypothetical protein
67	ZNDK_pg_47	S	pseudogene of hypothetical protein
68	ZNDK_pg_56	S	pseudogene of YbaB/EbfC family DNA-binding protein

69	ZNDK_pg_57	S	pseudogene of glutamine amidotransferase
70	ZNDK_pg_58	S	pseudogene of single-strand DNA-binding protein
71	ZNDK_pg_59	S	pseudogene of hypothetical protein
72	ZNDK_pg_62	S	pseudogene of hypothetical protein
73	ZNDK_pg_63	S	pseudogene of conserved hypothetical protein
74	ZNDK_pg_64	S	pseudogene of hypothetical protein
75	ZNDK_pg_65	S	pseudogene of conjugal transfer system, relaxase TraI
76	ZNDK_pg_71	S	pseudogene of membrane-associated HD superfamily hydrolase
77	ZNDK_pg_76	S	pseudogene of nitronate monooxygenase
78	ZNDK_pg_79	S	pseudogene of cell division protein FtsK
79	ZNDK_pg_80	S	pseudogene of DNA-binding protein
80	ZNDK_pg_81	S	pseudogene of hypothetical protein
81	ZNDK_pg_83	S	pseudogene of conserved hypothetical protein
82	ZNDK_pg_84	S	pseudogene of F0F1 ATP synthase subunit I
83	ZNDK_pg_10	T	pseudogene of sigma-54-dependent Fis family transcriptional regulator
84	ZNDK_pg_27	T	pseudogene of chemotaxis protein CheZ
85	ZNDK_pg_60	U	pseudogene of putative type IV secretion system component virB4
86	ZNDK_pg_25	V	pseudogene of type I restriction-modification system subunit R

* The NOG categories are as follows.
[J] Translation, ribosomal structure and biogenesis
[L] Replication, recombination and repair
[D] Cell cycle control, cell division, chromosome partitioning
[V] Defense mechanisms
[T] Signal transduction mechanisms
[N] Cell motility
[U] Intracellular trafficking, secretion, and vesicular transport
[C] Energy production and conversion
[G] Carbohydrate transport and metabolism
[E] Amino acid transport and metabolism
[F] Nucleotide transport and metabolism
[H] Coenzyme transport and metabolism
[P] Inorganic ion transport and metabolism
[Q] Secondary metabolites biosynthesis, transport and catabolism
[S] Function unknown

Table S9. Amino acid biosynthetic pathways in the genomes of symbionts associated with *Trichonympha* protists.

	Desulfovibrio phylotype ZnDsv-02	'*Ca*. Desulfovibrio trichonymphae' Rs-N31	'*Ca*. Endomicrobium trichonymphae' Rs-D17	'*Ca*. Adiutrix intracellularis' Adiu1 [5]
Alanine	+ (transporter)	+	+	+ (transporter)
Arginine	+	+	+	+
Asparagine	+	+	-	- (transporter?)
Aspartic acid	+	+	+	+
Cysteine	+	+	-	+
Glutamine	+	+	-	+
Glutamic acid	+	+	+	+
Glycine	+	+	+	+
Histidine	+	+	+	+
Isoleucine	+	+	+	+
Leucine	+	+	+	+
Lysine	+	+	+	+
Methionine	+	+ (transporter)	+	+
Phenylalanine	+	+	+	+
Proline	+	+	- (transporter)	+
Serine	+	+	- (transporter)	+
Threonine	+	+ (transporter?)	+	+
Tryptophan	+	+	+	+
Tyrosine	+	+	+	+
Valine	+	+	+	+

Table S10. Cofactor biosynthetic pathways in the genomes of symbionts associated with *Trichonympha* protists.

	Desulfovibrio phylotype ZnDsv-02	'*Ca*. Desulfovibrio trichonymphae' Rs-N31	'*Ca*. Endomicrobium trichonymphae' Rs-D17	'*Ca*. Adiutrix intracellularis' Adiu1 [5]
FAD	+	+	+	+
Riboflavin	+	+	+	+
THF	+	+	+	- (transporter?)
Thiamin-PP	+	+	+	+
NAD	+	+	+	+
NADP	+	+	+	+
Pantothenate	+	+	+	- (transporter?)
CoA	+	+	+	+
SAM	+	+	+	+
Heme	+	+	-	+
Siroheme	+	+	-	+
Cobalamin	-	+	- (transporter)	+ (transporter)
Biotin	-	-	+	- (transporter?)
Lipoate	-	-	+	-
Menaquinone	+	+	-	+

Table S11. Repertoire of genes involved in hydrogen metabolism in *Desulfovibrio* phylotype ZnDsv-02, '*Ca*. Desulfovibrio trichonymphae' phylotype Rs-N31 and their free-living relatives (modified from [9]).

	Desulfovibrio phylotype ZnDsv-02	'*Ca*. Desulfovibrio trichonymphae' Rs-N31	*D. fairfieldensis* CCUG 45958	*D. desulfuricans* ATCC 27774
periplasmic [NiFe] Hase	1	1	2	2
periplasmic [FeFe] Hase			1	1
Ech-Hase		1	1	1
Coo-Hase	1	1	1	1
cytoplasmic [FeFe] Hase				
periplasmic Fdh			2	2
cytoplasmic Hdr complex	1	1	1	1
lactate dehydrogenase	2		2	3
cytochrome oxidase			1	1
$TpIc_3$	1	1	1	1
Dsr	1	1	1	1
Qmo	1	1	1	1
Hmc	1	1		
Qrc				
Tmc				1
Nhc			1	1
Ohc				
Nrf	1	1	1	1
Rnf/Nqr/Nuo			1	2
Frd	1	1	2	1

Table S12. Genes involved in oxygen resistance of *Desulfovibrio* phylotype ZnDsv-02, '*Ca* . Desulfovibrio trichonymphae' phylotype Rs-N31 and their free-living relatives. Pseudogenes are shown in blue.

Desulfovibrio phylotype ZnDsv-02	'*Ca* . Desulfovibrio trichonymphae' Rs-N31	*Desulfovibrio fairfieldensis* CCUG 45958	*Desulfovibrio desulfuricans ATCC 27774*
-	-	Dfi_0012 cytochrome c oxidase assembly protein	Ddes_2195 cytochrome d ubiquinol oxidase, subunit II
-	-	Dfi_0013 cytochrome d ubiquinol oxidase subunit I	Ddes_2196 cytochrome bd ubiquinol oxidase subunit I
-	-	Dfi_0385 rubrerythrin	Ddes_0633 rubrerythrin
ZNDK_1226 rubrerythrin (NADH peroxidase)	RSDT_0752 rubrerythrin (NADH peroxidase)	Dfi_0571 rubrerythrin	Ddes_0897 rubrerythrin
-	RSDT_pg_146 pseudogene of catalase	Dfi_0585 Catalase	Ddes_1494 catalase
-	-	Dfi_0861 rubredoxin	Ddes_1386 rubredoxin-type Fe(Cys)4 protein
ZNDK_0185 redoxin-domain protein	-	Dfi_1042 redoxin	Ddes_1441 redoxin domain protein
ZNDK_0339 thioredoxin	RSDT_0325 thioredoxin	Dfi_1198 thioredoxin	Ddes_1199 thioredoxin
ZNDK_0338 NADPH-dependent thioredoxin reductase	RSDT_0324 NADPH-dependent thioredoxin reductase	Dfi_1197 thioredoxin reductase	Ddes_1200 thioredoxin reductase
-	-	Dfi_2053 cytochrome c551 peroxidase	-
-	-	Dfi_2074 superoxide dismutase	Ddes_0538 superoxide dismutase copper/zinc binding
ZNDK_1172 desulfoferrodoxin	RSDT_0359 desulfoferrodoxin	Dfi_2204 desulfoferrodoxin	Ddes_2010 desulfoferrodoxin
ZNDK_1173 rubredoxin	RSDT_0360 rubredoxin	Dfi_2205 rubredoxin	Ddes_2011 rubredoxin-type Fe(Cys)4 protein
ZNDK_1174 MBL fold metallo-hydrolase	RSDT_pg_062 pseudogene of rubredoxin-oxygen oxidoreductase	Dfi_2206 MBL fold metallo-hydrolase	Ddes_2012 beta-lactamase domain protein
-	-	Dfi_2452 thioredoxin	Ddes_0117 thioredoxin domain protein
-	-	Dfi_2455 thioredoxin reductase	Ddes_0114 thioredoxin reductase
-	RSDT_pg_138 pseudogene of dye-decolorishing (DyP) peroxidase family protein	-	Ddes_0076 Dyp-type peroxidase family

Table S13. Type IV secretion system and predicted properties of the VirB proteins found in the ZnDsv-02 genome (Created based on [10]). Functions related to host cell attachment are shown in red.

T4SS component	gene	localization	function
VirB1	ZNDK_1206	Periplasm	Transglycosylase for the assembly of pilus
VirB2	ZNDK_1132	Outer membrane and Extracellular surface	Major pilus component
VirB3	ZNDK_1133	Inner membrane	Unkonown
VirB4	ZNDK_pg_60	Inner membrane	ATPase, essential for secretion
VirB5	ZNDK_1134	Extracellular surface	Minor pilus component, candidate adhesin
VirB6	ZNDK1135	Inner membrane	Candidate pore former
VirB7	-	Periplasm	Core complex, essential for secretion
VirB8	ZNDK_1136	Inner membrane	Candidate pore former
VirB9	ZNDK_1137	Periplasm	Core complex
VirB10	ZNDK_1139	Inner and outer membrane	Core complex
VirB11	ZNDK_1130	Inner membrane	ATPase
VirD4	ZNDK_1143	Inner membrane	ATPase

Table S14. *Desulfovibrio* bacteria used as outgroups in the phylogenetic trees shown in Fig. 5.

strain	origin of samples	accession number
Desulfovibrio sp. KRS1	termite gut (*Reticulitermes santonensis*)	X93146
RsaHf324	termite gut (*Reticulitermes santonensis*)	AY571497
D. intestinalis KMS2	termite gut (*Mastotermes darwiniensis*)	Y12254
Desulfovibrio sp. G11	bovine rumen fluid (*Bos taurus*)	CP023415
D. desulfuricans ATCC 27774	sheep rumen (*Ovis aries*)	NC_011883
D. fairfieldensis CCUG 45958	human oral	CP014229
D. piger FI11049	human faces	LT630450
MgMjR-025	termite gut (*Macrotermes gilvus*)	AB234535
BCf11-19	termite gut (*Coptotermes formosanus*)	AB062810
Tc-45	termite gut (*Termes comis*)	AB189689
Nt2-001	termite gut (*Nasutitermes takasagoensis*)	AB255925
Lawsonia intracellularis PHE/MN1-00	pig intestinal tissue	NC_008011
RPK-66	termite gut (*Reticulitermes* sp. RPK)	AB192288
290cost002-P3L-618	termite gut (*Nasutitermes* sp.)	EF454896
MgMjD-050	termite gut (*Macrotermes gilvus*)	AB234530
290cost002-P3L-532	termite gut (*Nasutitermes* sp.)	EF454852
MgMjD-084	termite gut (*Macrotermes gilvus*)	AB234532
D. vulgaris str. Miyazaki F	degraded paddy field	NC_011769
D. termitidis HI1	termite gut (*Heterotermes indicola*)	X87409
D. vulgaris str. Hildenborough	clay soil	NC_011769
D. vulgaris DP4	clay soil	NC_008751
D. vulgaris RCH1	aquifer	NC_017310

Table S15. Number and frequency of *Desulfovibrio* ASVs detected by 16S rRNA amplicon sequencing analysis of the bacterial gut microbiota of various termites and cockroaches

(sub) family of host insects	species of host insects*	host code for ASVs	total number of reads	total number of ASVs	total number of *Desulfovibrio* reads	total number of *Desulfovibrio* ASVs	frequency of total *Desulfovibrio* reads	number of *Desulfovibrio* ASVs belonging to Termite Cluster 1	number of *Desulfovibrio* reads belonging to Termite Cluster 1	frequency of *Desulfovibrio* reads belonging to Termite Cluster 1
Ectobiidae	*Blatella germanica*	Bg	10209	165	1577	19	15.4%	0	0	0.0%
Blattellidae	*Symploce gigas*	SYg	726638	656	26510	31	3.6%	0	0	0.0%
Blattidae	*Periplaneta americana*	PPam	19338	329	972	19	5.0%	0	0	0.0%
Blattidae	*Periplaneta japonica*	PPja	39196	416	4196	27	10.7%	0	0	0.0%
Blaberidae	*Opisthoplatia orientalis*	OPo	15621	287	913	15	5.8%	0	0	0.0%
Blaberidae	*Salganea taiwanensis*	SGt	15314	367	69	3	0.5%	0	0	0.0%
Blaberidae	*Panesthia angustipennis*	Pa	10080	224	79	3	0.8%	0	0	0.0%
Cryptocercidae	*Cryptocercus clevelandi*	CCc	47548	299	260	5	0.5%	0	0	0.0%
Cryptocercidae	*Cryptocercus kyebangensis*	CCk	41438	356	415	5	1.0%	0	0	0.0%
Cryptocercidae	*Cryptocercus punctulatus*	CCp	13446	188	207	4	1.5%	0	0	0.0%
Mastotermitidae	*Mastotermes darwiniensis*	Md2	23830	202	0	0	0.0%	0	0	0.0%
Archotermopsidae	*Archotermopsis wroughtoni*	APK	29448	332	116	3	0.4%	1	34	0.1%
Archotermopsidae	*Hodotermopsis sjoestedti*	Hs3	25985	420	680	3	2.6%	1	623	2.4%
Archotermopsidae	*Hodotermopsis* sp.	HsK	27094	304	120	3	0.4%	1	7	0.0%
Archotermopsidae	*Zootermopsis nevadensis*	Zn3	33112	345	2013	8	6.1%	2	1647	5.0%
Stolotermitidae	*Porotermes adamsoni*	PRa	47714	365	225	2	0.5%	1	131	0.3%
Stolotermitidae	*Stolotermes victoriensis*	Sv	28305	229	511	5	1.8%	0	0	0.0%
Kalotermitidae	*Cryptotermes cavifrons*	Cc3	38071	228	407	6	1.1%	0	0	0.0%
Kalotermitidae	*Ceratokalotermes* sp.	CKsX	27579	201	896	7	3.2%	2	121	0.4%
Kalotermitidae	*Glyptotermes fuscus*	Gf2	62620	230	866	6	1.4%	1	126	0.2%
Kalotermitidae	*Glyptotermes satsumensis*	Gs	25520	249	201	5	0.8%	1	26	0.1%
Kalotermitidae	*Neotermes* sp.	NinX	45742	249	146	4	0.3%	1	5	0.0%
Kalotermitidae	*Neotermes koshunensis*	Nk	18688	157	0	0	0.0%	0	0	0.0%
Kalotermitidae	*Neotermes koshunensis*	Nk2	24606	161	0	0	0.0%	0	0	0.0%
Kalotermitidae	*Neotermes koshunensis*	Nk3	26658	206	173	5	0.6%	0	0	0.0%
Rhinotermitidae	*Parrhinotermes microdentiformis*	PRRm	38141	235	151	3	0.4%	0	0	0.0%
Rhinotermitidae	*Parrhinotermes microdentiformis*	PRRm2	45538	227	297	7	0.7%	1	19	0.0%
Rhinotermitidae	*Parrhinotermes sp.*	PRCx	44085	422	583	9	1.3%	1	93	0.2%
Rhinotermitidae	*Schedorhinotermes* sp. 1	SCaX	26410	383	631	16	2.4%	2	156	0.6%
Rhinotermitidae	*Schedorhinotermes medioobscurus*	SCm	33547	315	317	8	0.9%	2	99	0.3%
Rhinotermitidae	*Schedorhinotermes* sp. 2	SCmX	37400	349	747	13	2.0%	1	85	0.2%
Rhinotermitidae	*Schedorhinotermes sarawakensis*	SCs	25192	456	1458	18	5.8%	3	345	1.4%
Rhinotermitidae	*Prorhinotermes japonicus*	PRHj	33775	243	72	3	0.2%	0	0	0.0%
Rhinotermitidae	*Prorhinotermes inopinatus*	Pi	70219	224	170	3	0.2%	0	0	0.0%
Rhinotermitidae	*Coptotermes formosanus*	Cf3	34712	177	24	2	0.1%	0	0	0.0%
Rhinotermitidae	*Coptotermes curvignathus*	COPc	29422	219	89	4	0.3%	0	0	0.0%
Rhinotermitidae	*Heterotermes cf. paradoxus*	HETp	48603	320	49	2	0.1%	0	0	0.0%
Rhinotermitidae	*Heterotermes tenuior*	HETt	44595	299	111	3	0.2%	1	53	0.1%
Rhinotermitidae	*Reticulitermes amamianus*	Ra	40771	341	4958	3	12.2%	2	4946	12.1%
Rhinotermitidae	*Reticulitermes miyatakei*	Rm	40339	400	6831	4	16.9%	3	6802	16.9%
Rhinotermitida	*Reticulitermes* sp. RPK	RPK2	51927	377	4622	4	8.9%	1	4536	8.7%
Rhinotermitidae	*Reticulitermes speratus*	RsTz2	49601	486	3122	8	6.3%	1	2959	6.0%
Rhinotermitidae	*Reticulitermes yaeyamanus*	Ry	32991	346	1618	5	4.9%	1	1579	4.8%
Rhinotermitidae	*Termitogeton planus*	TGp	56778	365	263	9	0.5%	1	14	0.0%
Termitidae	*Amitermes longignathus*	Alo	31710	730	424	7	1.3%	0	0	0.0%
Macrotermitinae	*Amitermes laurensis*	Ala	16171	385	162	5	1.0%	0	0	0.0%
Macrotermitinae	*Dicuspiditermes nemorosus*	DCn	18012	422	180	4	1.0%	0	0	0.0%
Macrotermitinae	*Dicuspiditermes nemorosus*	DCn2	13838	373	108	6	0.8%	0	0	0.0%
Apicotermitinae	*Drepanotermes rubriceps*	DRr	28123	426	86	1	0.3%	0	0	0.0%
Amitermitinae	*Globitermes globosus*	GBg	28847	334	62	2	0.2%	0	0	0.0%
Amitermitinae	*Homallotermes eleanorae*	HMe	21071	357	402	10	1.9%	0	0	0.0%
Amitermitinae	*Microcerotermes* sp. M1	M1PT4b	26400	337	325	7	1.2%	0	0	0.0%
Termitinae	*Microcerotermes crassus*	McPP3	42885	244	0	0	0.0%	0	0	0.0%
Termitinae	*Macrognathotermes errator*	MGe	22694	405	149	4	0.7%	0	0	0.0%
Termitinae	*Microcerotermes dubius*	MCd	18886	161	49	1	0.3%	0	0	0.0%
Termitinae	*Prohamitermes mirabilis*	PHm	25713	604	234	6	0.9%	0	0	0.0%
Termitinae	*Prohamitermes mirabilis*	PHm2	31016	532	1018	19	3.3%	0	0	0.0%
Termitinae	*Pericapritermes nitobei*	Pn	29581	688	263	9	0.9%	0	0	0.0%
Termitinae	*Pericapritermes dolichocephalus*	Pd	12161	415	88	6	0.7%	0	0	0.0%
Termitinae	*Procapritermes* sp.	PpX	26000	561	607	16	2.3%	0	0	0.0%
Termitinae	*Procapritermes setiger*	PRCx	16781	383	291	4	1.7%	0	0	0.0%
Termitinae	*Sinocapritermes mushae*	Sim	39095	576	376	7	1.0%	0	0	0.0%
Termitinae	*Termes comis*	Tc2	18431	567	588	11	3.2%	0	0	0.0%
Termitinae	*Termes comis*	TcM	20786	565	221	7	1.1%	0	0	0.0%
Termitinae	*Termes comis*	TcM2	12337	497	141	6	1.1%	0	0	0.0%
Termitinae	*Termes rostratus*	Trr	18478	644	300	8	1.6%	0	0	0.0%
Termitinae	*Macrotermes gilvus*	MgMjD2	13349	271	277	7	2.1%	0	0	0.0%
Termitinae	*Macrotermes malaccensis*	Mm	32146	463	969	13	3.0%	0	0	0.0%
Termitinae	*Odontotermes formosanus*	Of	26417	212	671	14	2.5%	0	0	0.0%
Termitinae	*Hospitalitermes* sp. 2	HOSb	25690	405	583	9	2.3%	0	0	0.0%
Nasutitermitinae	*Hospitalitermes* sp. 1	HOSp	25539	422	1156	15	4.5%	0	0	0.0%
Nasutitermitinae	*Hospitalitermes medioflavus*	HOSm	37542	406	361	9	1.0%	0	0	0.0%
Nasutitermitinae	*Leucopitermes leucops*	LEUl	21675	288	246	5	1.1%	0	0	0.0%
Nasutitermitinae	*Longipeditermes longipes*	LONl	51955	376	539	11	1.0%	0	0	0.0%
Nasutitermitinae	*Nasutitermes dimorphus*	Nd	38092	168	223	3	0.6%	0	0	0.0%
Nasutitermitinae	*Nasutitermes longinasus*	Nl	44514	392	294	11	0.7%	0	0	0.0%
Nasutitermitinae	*Nasutitermes longinasus*	Nl2	94413	590	1216	14	1.3%	0	0	0.0%
Nasutitermitinae	*Nasutitermes takasagoensis*	Nt2	26730	180	258	5	1.0%	0	0	0.0%
Nasutitermitinae	*Nasutitermes triodiae*	Ntr	36654	289	120	2	0.3%	0	0	0.0%
Nasutitermitinae	*Nasutitermes takasagoensis*	NtTw	76687	477	1101	12	1.4%	0	0	0.0%
Nasutitermitinae	*Oriensubulitermes inanis*	ORi	29709	782	874	20	2.9%	0	0	0.0%
Nasutitermitinae	*Speculitermes* sp.	SPEx	25327	287	1117	8	4.4%	0	0	0.0%

* Host species, in which the frequncy of *Desulfovibrio* sequence reads exceeded 5.0%, are maked.

References for Supplementary Tables

1 Amann RI, Binder BJ, Olson RJ, Chisholm SW, Devereux R, Stahl DA. Combination of 16S rRNA-targeted oligonucleotide probes with flow cytometry for analyzing mixed microbial populations. Appl Environ Microbiol. 1990; 56: 1919–1925.

2 Sato T, Hongoh Y, Noda S, Hattori S, Ui S, Ohkuma M. *Candidatus* Desulfovibrio trichonymphae, a novel intracellular symbiont of the flagellate *Trichonympha agilis* in termite gut. Environ Microbiol. 2009; 11: 1007–1015.

3 Manz W, Eisenbrecher M, Neu TR, Szewzyk U. Abundance and spatial organization of Gram-negative sulfate-reducing bacteria in activated sludge investigated by in situ probing with specific 16S rRNA targeted oligonucleotides. FEMS Microbiol Ecol. 1998; 25: 43–61.

4 Ikeda-Ohtsubo W, Desai M, Stingl U, Brune A. Phylogenetic diversity of 'Endomicrobia' and their specific affiliation with termite gut flagellates. Microbiology. 2007; 153 : 3458–3465.

5 Ikeda-Ohtsubo W, Strassert JFH, Köhler T, Mikaelyan A, Gregor I, McHardy AC, et al. '*Candidatus* Adiutrix intracellularis', an endosymbiont of termite gut flagellates, is the first representative of a deep-branching clade of *Deltaproteobacteria* and a putative homoacetogen. Environ Microbiol. 2016; 18: 2548–2564.

6 Klindworth A, Pruesse E, Schweer T, Peplies J, Quast C, Horn M, et al. Evaluation of general 16S ribosomal RNA gene PCR primers for classical and next-generation sequencing-based diversity studies. Nucleic Acids Res. 2013; 41: e1.

7 Sato T, Kuwahara H, Fujita K, Noda S, Kihara K, Yamada A, et al. Intranuclear verrucomicrobial symbionts and evidence of lateral gene transfer to the host protist in the termite gut. ISME J. 2014; 8: 1008–1019.

8 Tai V, Carpenter KJ, Weber PK, Nalepa CA, Perlman SJ, Keeling PJ. Genome evolution and nitrogen fixation in bacterial ectosymbionts of a protist inhabiting wood-feeding cockroaches. Appl Environ Microbiol. 2016; 82: 4682–4695.

9 Pereira IAC, Ramos AR, Grein F, Marques MC, da Silva SM, Venceslau SS. A comparative genomic analysis of energy metabolism in sulfate reducing bacteria and archaea. Front Microbiol. 2011; 2: 69.

10 Wallden K, Rivera-Calzada A, Waksman G. Type IV secretion systems: versatility and diversity in function. Cell Microbiol. 2010; 12: 1203–1212.
