# Supplementary material for: Parallel reductive genome evolution in Desulfovibrio ectosymbionts independently acquired by Trichonympha protists in the termite gut
Source: ISME J. 2020 Jun 1;14(9):2288–301. doi: 10.1038/s41396-020-0688-1 (PMC7608387; doi:10.1038/s41396-020-0688-1)
Supplement: Supplementary file 3 — Supplementary Figures [file 41396_2020_688_MOESM3_ESM.pdf]

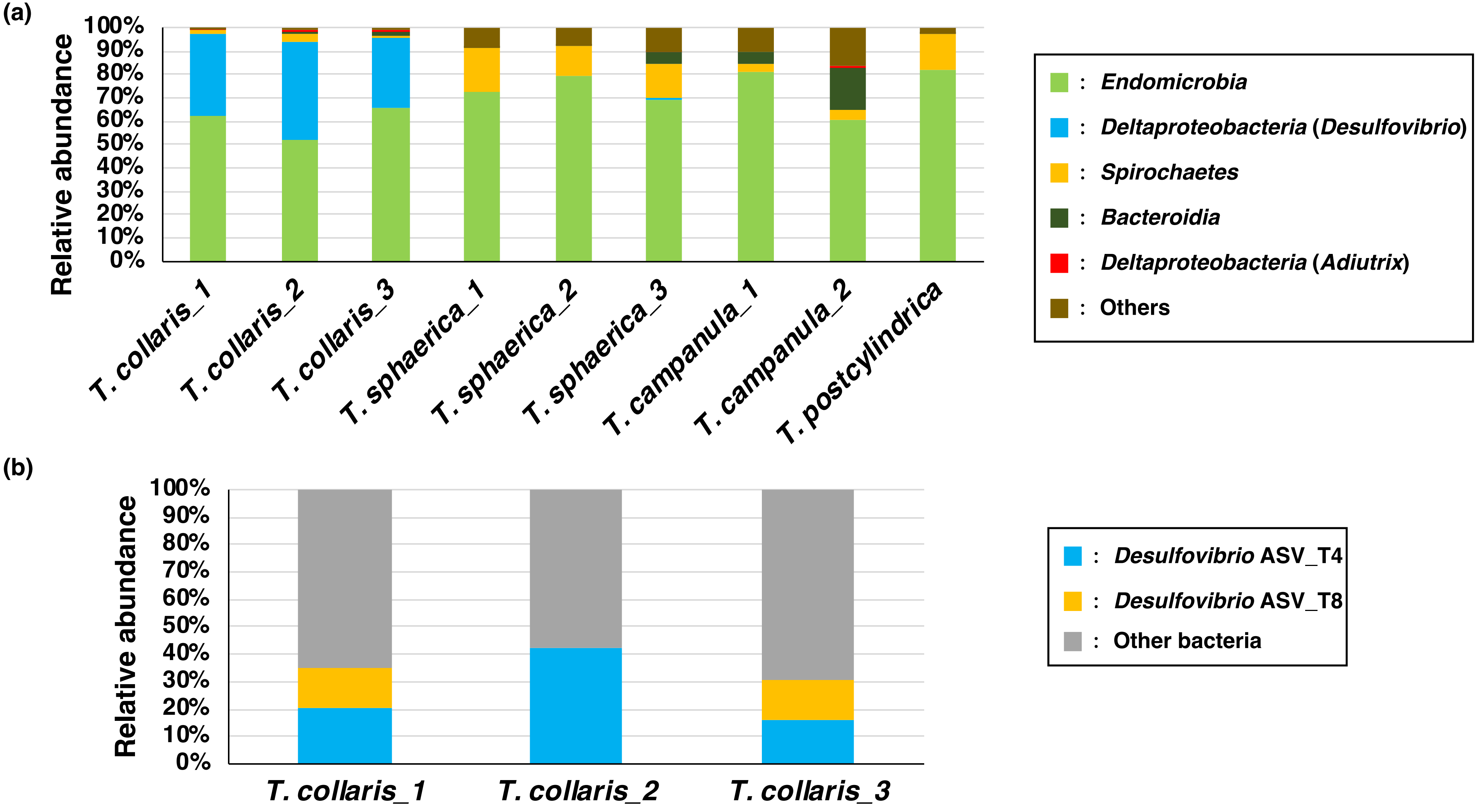

Figure S1. (**a**) Taxonomic composition of bacteria based on 16S rRNA amplicon sequencing analysis associated with single cells of four *Trichonympha* species from the gut of *Zootermopsis nevadensis*. *Desulfovibrio* were detected in all three *T. collaris* cells and one of three *T. sphaerica* cells. The frequency of *Desulfovibrio* reads from the *T. sphaerica* cell was 0.7%, whereas that in the *T. collaris* cells was >30%. As *Desulfovibrio* cells were detected only in *T. collaris* cells by FISH analysis using probe DSV698, the *Desulfovibrio* reads recovered from the *T. sphaerica* cell were likely contaminants from the gut luminal fluid. (**b**) Relative abundance of *Desulfovibrio* ASVs associated with single cells of *T. collaris*.

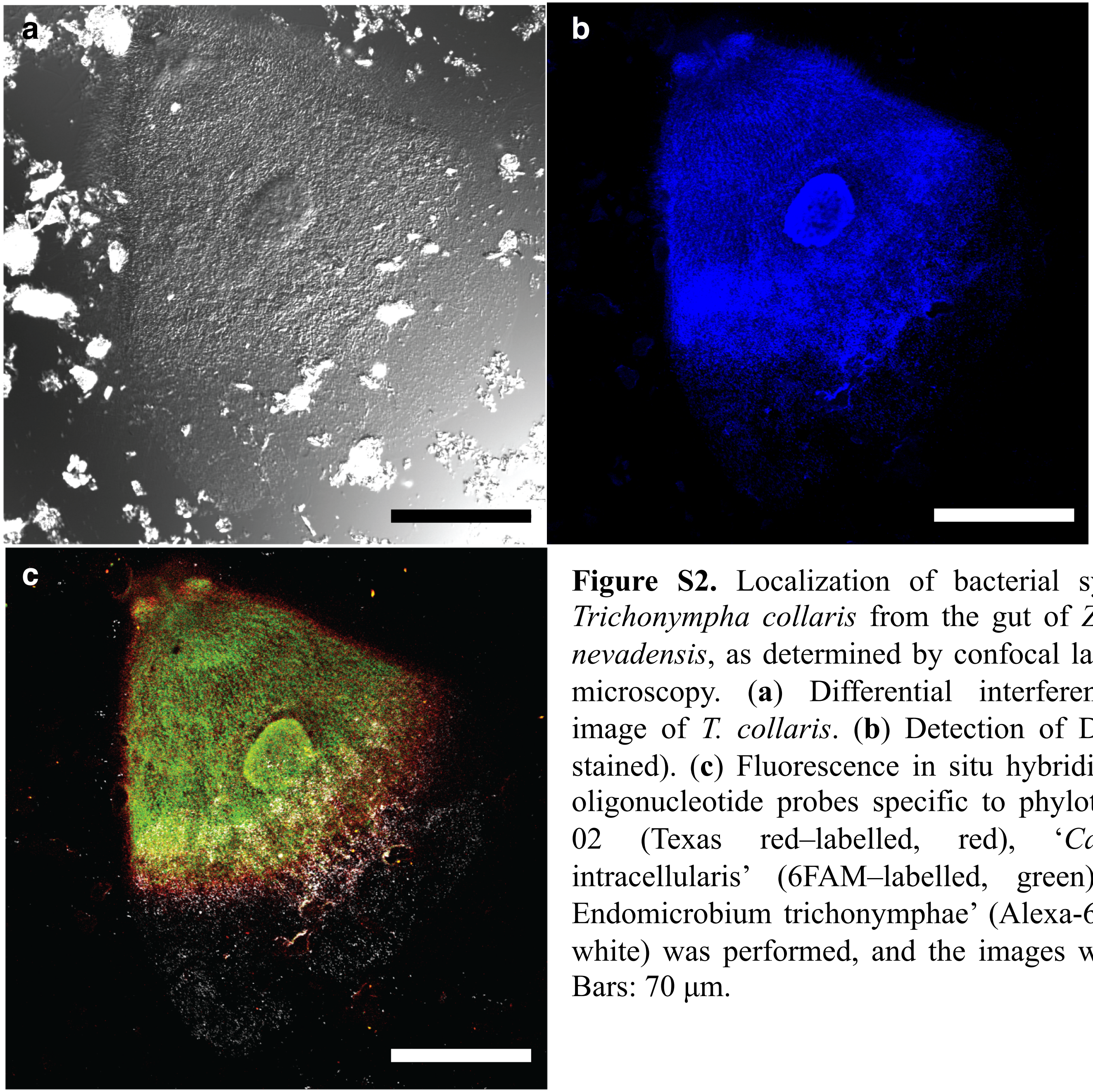

Figure S2. Localization of bacterial symbionts of *Trichonympha collaris* from the gut of *Zootermopsis nevadensis*, as determined by confocal laser scanning microscopy. (**a**) Differential interference contrast image of *T. collaris*. (**b**) Detection of DNA (DAPI-stained). (**c**) Fluorescence in situ hybridization using oligonucleotide probes specific to phylotype ZnDsv-02 (Texas red–labelled, red), '*Ca*. Adiutrix intracellularis' (6FAM–labelled, green) and '*Ca*. Endomicrobium trichonymphae' (Alexa-647–labelled, white) was performed, and the images were merged. Bars: 70 µm.

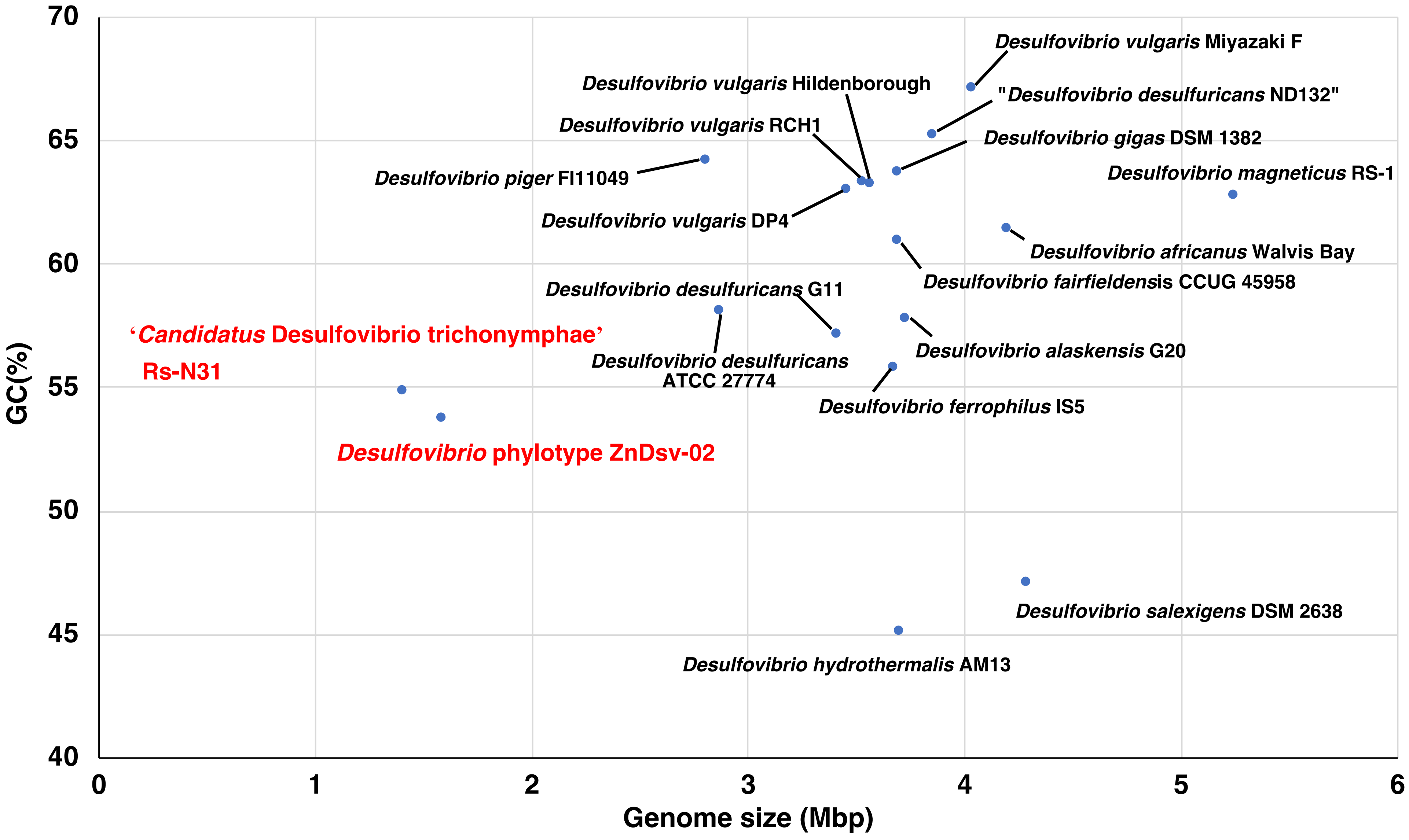

Figure S3. Comparison of G+C content among the genomes of *Desulfovibrio* phylotype ZnDsv-02 and other *Desulfovibrio* species.

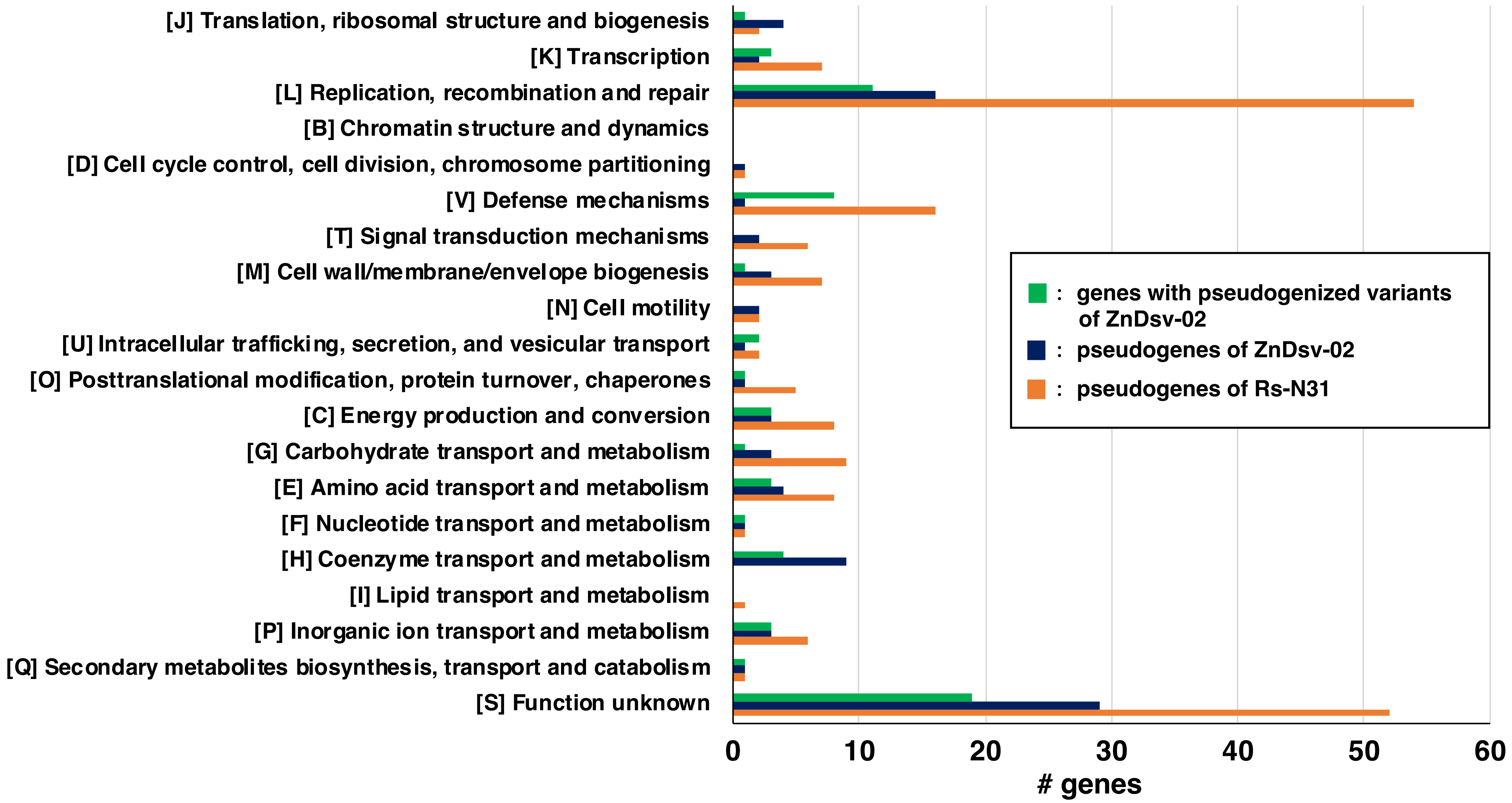

Figure S4. Comparison of the number of genes classified into non-supervised orthologous groups among genes with pseudogenized variants of *Desulfovibrio* phylotype ZnDsv-02, pseudogenes of ZnDsv-02, and pseudogenes of '*Ca*. Desulfovibrio trichonymphae' phylotype Rs-N31.

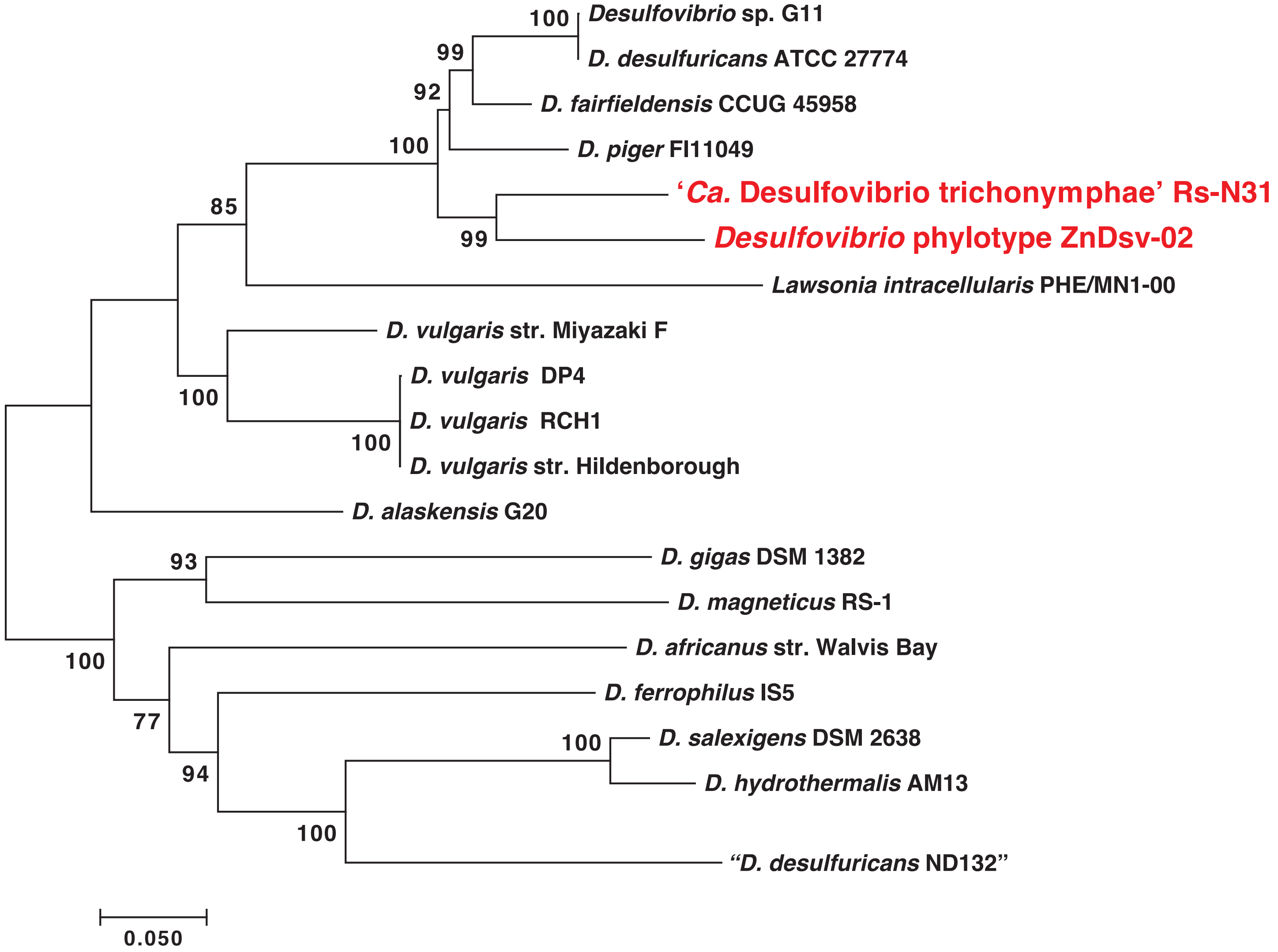

Figure S5. Maximum-likelihood tree based on the concatenated amino acid sequences of 30 ribosomal proteins. Ribosomal proteins L1, L2, L4, L5, L6, L10, L11, L13, L14, L15, L16, L18, L22, L23, L24, L25, L29, S2, S3, S5, S7, S8, S9, S10, S11, S12, S13, S15, S17, and S19 were used. A total of 4 056 aligned amino acid sites were used in the analysis. The LG+G+I amino acid substitution model was used. Bootstrap analysis was performed with 500 resamplings. Bootstrap confidence values >70% are shown.

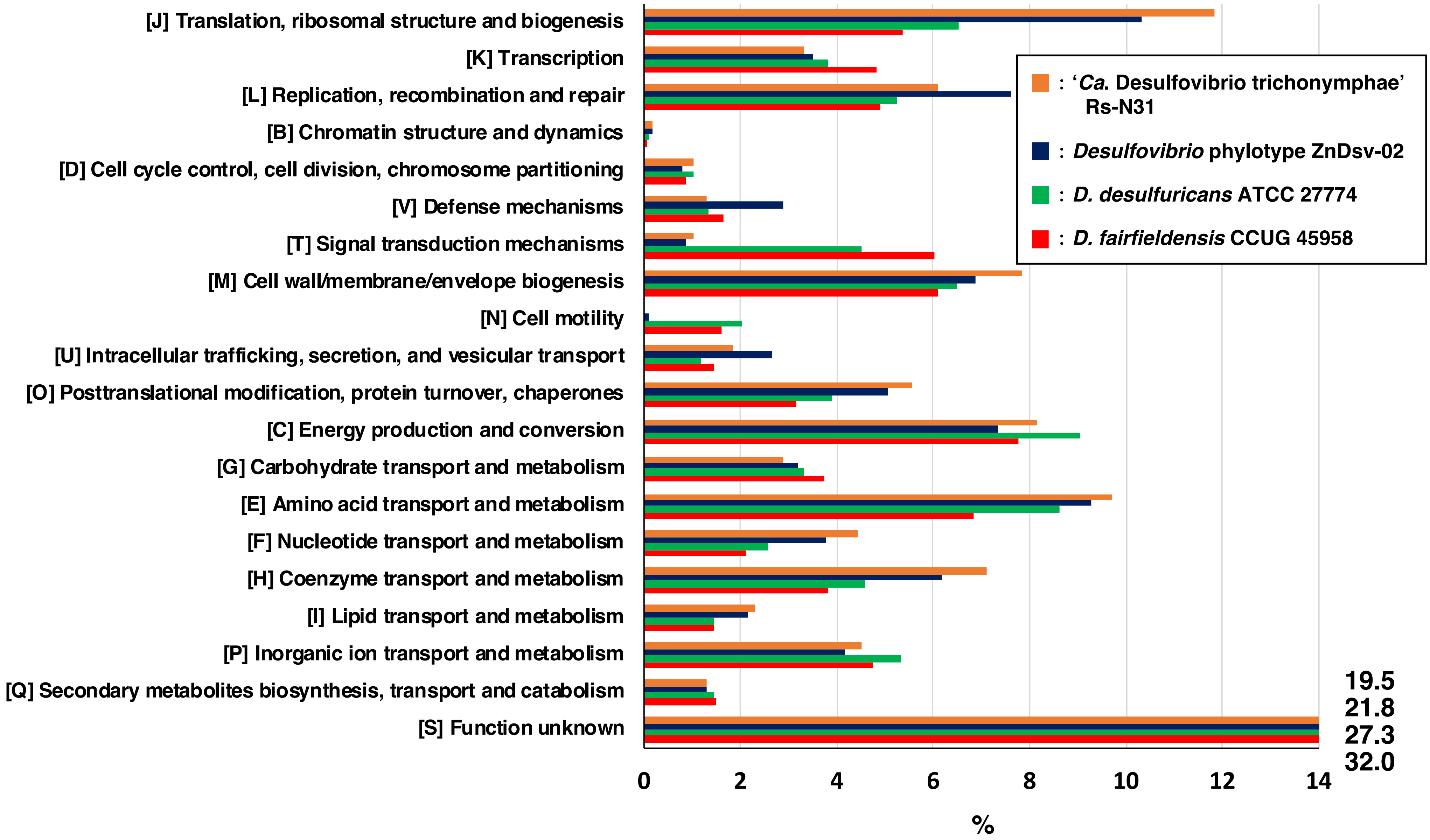

Figure S6. Comparison of the ratio of genes classified into non-supervised orthologous groups among the genomes of *Desulfovibrio* phylotype ZnDsv-02, '*Ca*. Desulfovibrio trichonymphae' phylotype Rs-N31 and the free-living relatives *Desulfovibrio fairfieldensis* and *Desulfovibrio desulfuricans*.

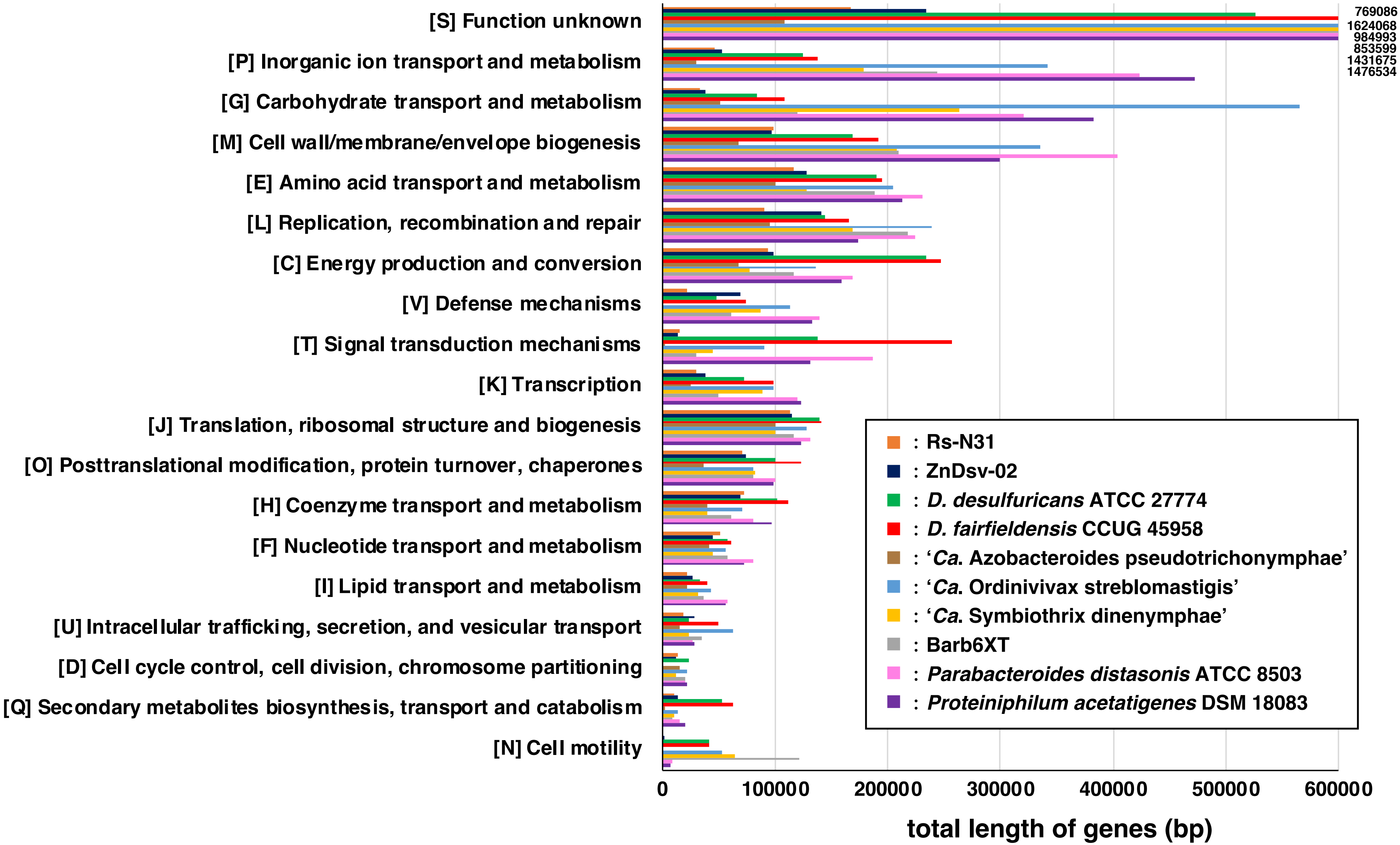

Figure S7. Comparison of the total length of genes classified into non-supervised orthologous groups among the genomes of the *Desulfovibrio* ectosymbionts ZnDsv-02 and Rs-N31, free-living *Desulfovibrio* species, *Bacteroidales* ectosymbionts ('*Ca*. Ordinivivax streblomastigis', '*Ca*. Symbiothrix dinenymphae' and phylotype Barb6XT) and their free-living/endosymbiotic *Bacteroidales* relatives.

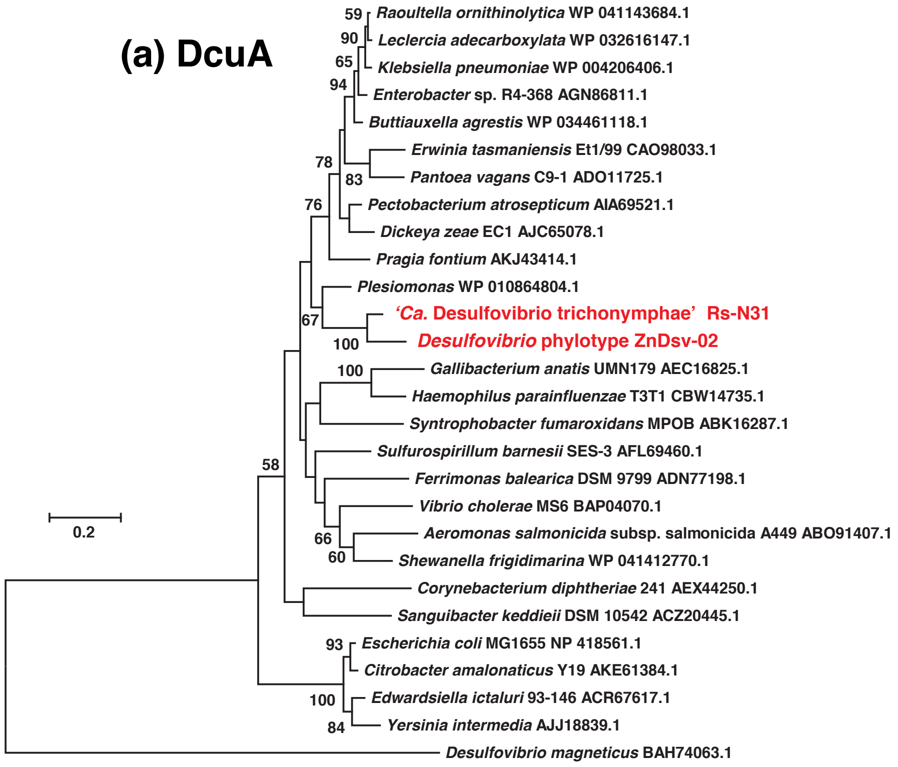

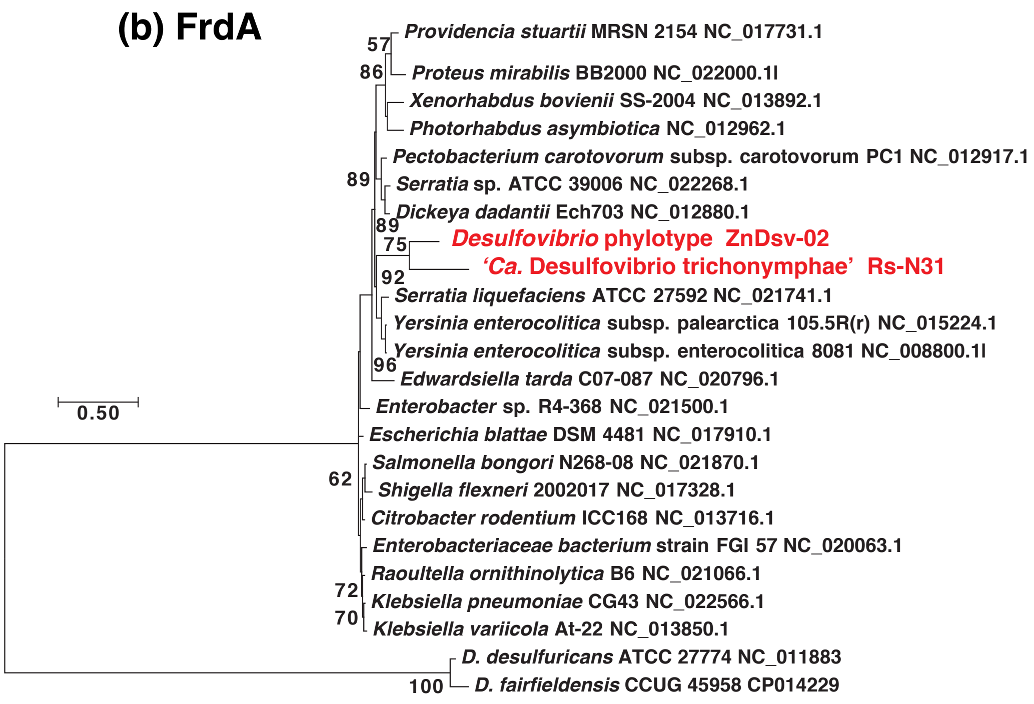

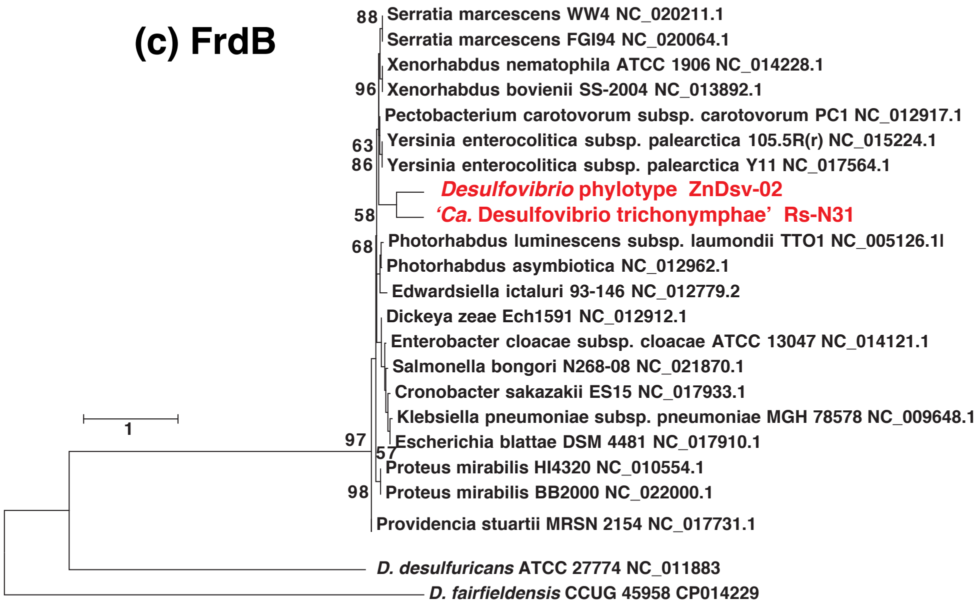

Figure S8. Maximum-likelihood trees of DcuA, FrdA and FrdB, based on the deduced amino acid sequences. (**a**) A total of 311 aligned amino acid sites were used. The LG+G amino acid substitution model was used. (**b**) A total of 355 aligned amino acid sites were used. The LG+G amino acid substitution model was used. (**c**) A total of 108 aligned amino acid sites were used. The Dayhoff+G amino acid substitution model was used. Bootstrap analysis was performed with 500 resamplings. Bootstrap confidence values >50% are shown.

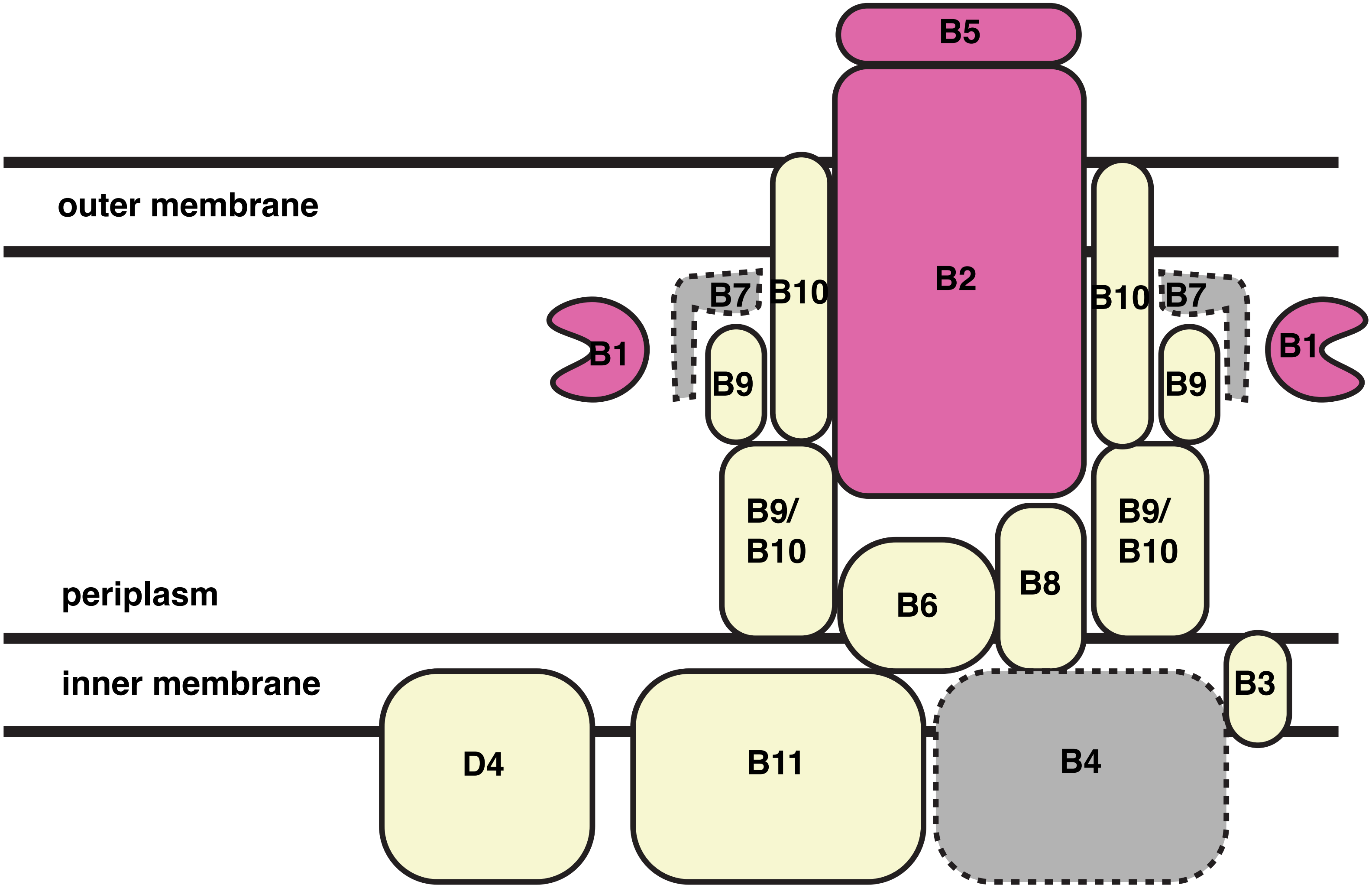

Figure S9. Type IV secretion system identified in the *Desulfovibrio* phylotype ZnDsv-02 genome. Genes encoding components in grey surrounded by dashed lines are missing or pseudogenized in the genome. Components shown in magenta are probably involved in cell-cell contact. Image was created based on a previous study [16].
